# Novel Synthesis Pathways for Highly Oxidative Iron Species: Generation, Stability, and Treatment Applications of Ferrate(IV/V/VI)

**DOI:** 10.1021/acs.est.2c09237

**Published:** 2023-02-16

**Authors:** Sean T. McBeath, Yi Zhang, Michael R. Hoffmann

**Affiliations:** † Linde Laboratories, California Institute of Technology, Pasadena, California 91125, United States; ‡ Department of Civil and Environmental Engineering, University of Massachusetts Amherst, Amherst, Massachusetts 01002, United States

**Keywords:** Electro-oxidation, electro-synthesis, ferrate, ozone, boron-doped diamond, nickel-doped antimony tin oxide

## Abstract

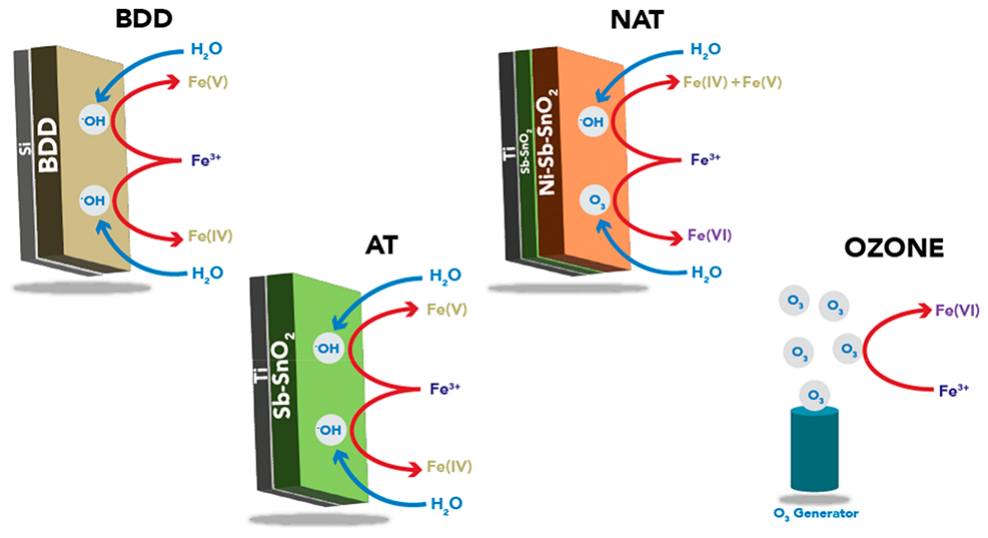

Difficulties arise related to the economy-of-scale and practicability in applying conventional water treatment technologies to small and remote systems. A promising oxidation technology better suited for these applications is that of electro-oxidation (EO), whereby contaminants are degraded via direct, advanced, and/or electrosynthesized oxidant-mediated reactions. One species of oxidants of particular interest includes ferrates (Fe(VI)/(V)/(IV)), where only recently has their circumneutral synthesis been demonstrated, using high oxygen overpotential (HOP) electrodes, namely boron-doped diamond (BDD). In this study, the generation of ferrates using various HOP electrodes (BDD, NAT/Ni–Sb–SnO_2_, and AT/Sb-SnO_2_) was investigated. Ferrate synthesis was pursued in a current density range of 5–15 mA cm^–2^ and initial Fe^3+^ concentrations of 10–15 mM. Faradaic efficiencies ranged from 11–23%, depending on operating conditions, with BDD and NAT significantly outperforming AT electrodes. Speciation tests revealed that NAT synthesizes both ferrate(IV/V) and ferrate(VI), while the BDD and AT electrodes synthesized only ferrate(IV/V) species. A number of organic scavenger probes were used to test the relative reactivity, including nitrobenzene, carbamazepine, and fluconazole, whereby ferrate(IV/V) was significantly more oxidative than ferrate(VI). Finally, the ferrate(VI) synthesis mechanism by NAT electrolysis was elucidated, where coproduction of ozone was found to be a key phenomenon for Fe^3+^ oxidation to ferrate(VI).

## Abstract 2

This manuscript presents a novel synthesis pathway for high oxidative iron species suitable for decentralized water treatment applications.

## Introduction

The prospect of electrochemical technologies in water treatment processes has been growing due to their favorability in various niche applications when compared to traditional technologies. In general, electrochemical technologies are suitable in nonconventional water treatment applications, in part due to their favorable economy-of-scale and relative simplicity. Electrochemical oxidation (electro-oxidation, EO), for example, is a promising alternative technology for small and decentralized system applications, as it can eliminate the chemical supply chain associated with conventional oxidation/disinfection options by generating chemicals on-site and on-demand.

Conventional EO processes, employing efficient nonactive electrode materials, typically proceed via two reaction pathways: (1) direct electron transfer (DET) at the electrode surface and (2) hydroxyl radical (^•^OH)-mediated oxidation. The latter reaction pathway is possible during EO when using high oxygen overpotential (HOP) electrode materials, such as boron-doped diamond (BDD) and other mixed metal oxides (MMO), which possess a greatly increased potential range (reduction and oxidation) of water stability (e.g., BDD: −1.25–2.3 V_SHE_^1^). Although the primary mechanism for pollutant degradation during EO processes has been attributed to ^•^OH-mediated oxidation,^2^ the reaction is limited at the electrode surface where reactive oxygen species are generated.^3^ A third EO reaction mechanism exists, however, whereby the electrosynthesis of residual chemical oxidants proceeds via ion oxidation at the electrode surface, resulting in pollutant degradation in the bulk water solution (e.g., not limited to the electrode surface). Some examples of electro-generated oxidant species include persulfate (*E*^0^ = 1.96 V_SHE_),^4^ peroxodiphosphate (*E*^0^ = 2.07 V_SHE_),^5^ and various reactive chlorine species^6,7^ when sulfate, phosphate, and chloride are present in the water matrix, respectively.

An additional group of powerful oxidants that has yet to receive the same attention, as it relates to circumneutral electrosynthesis for water treatment applications, is the generation of high oxidation state iron species known as ferrates. Ferrates are particularly well-suited for water treatment applications,^8−10^ as they are not known to form recalcitrant oxidation byproducts like chlorinated disinfection byproducts^11^ and their reduced products are nontoxic hydrolysis Fe^3+^ species, which have also been reported to effectively function as coagulant chemicals.^12−14^ Most commonly in water treatment practices, potassium ferrate (K_2_FeO_4_) is used to form aqueous ferrate(VI) (Fe(VI)/Fe^VI^O_4_^2–^), which is characteristically purple and has a high redox potential (*E*^0^ = 2.2 V_SHE_). However, lesser reported high oxidation state iron species also exist, namely ferrate(V) (Fe(V)/Fe^V^O_3_^3–^) and ferrate(IV) (Fe(IV)/Fe^IV^O_4_^4–^).^15,16^ Some studies have found that Fe(V) and Fe(IV) ferrate species yield degradation rates as much as 2–5 orders of magnitude greater than Fe(VI) for various organic pollutants, such as organosulfur and phenolic compounds, in high pH conditions.^16,17^ In general, much is still unknown about the various ferrate species and their reactivity and stability in circumneutral aqueous conditions.

While ferrates are conventionally synthesized using a wet chemical method by oxidation of Fe^3+^ in highly alkaline conditions,^15,18−20^ electrochemical^21,22^ and thermal chemical^22^ methods also exist under challenging and unstable conditions. More recently, evidence of circumneutral ferrate electrosynthesis has been reported through the use of BDD electrodes and Fe^2+^/Fe^3+^ precursors for water treatment applications.^23−29^ In these studies, however, no iron speciation was performed and ferrate was assumed to be in its most stable Fe(VI) form. Although BDD has been the preferred material for the circumneutral generation of ferrate to date, due to its aforementioned electrocatalytic properties, it also has several limitations. In addition to prohibitive costs,^30^ BDD electrodes require slow growth rates to yield high quality films, they are limited to substrates that are compatible with its growth conditions, and they are generally size-limited due to the chemical vapor deposition method by which they are synthesized.^31^ Another group of promising materials includes substoichiometric alternatives to platinum group metal oxides, known as Magnéli phase titanium oxides (Ti_*n*_O_2*n*–1_, 4 ≤ *n* ≤ 10),^32,33^ which also possess many favorable electrocatalytic properties for water treatment and ferrate synthesis. However, similar to BDD, their applications are somewhat limited due to a challenging production process requiring the reduction of TiO_2_ in high temperature conditions and pure H_2_.^34^ Another material considered as an HOP material is antimony-doped tin(IV) oxide (AT/Sb-SnO_2_), which has a comparatively facile and inexpensive preparation method by dip or brush coating and subsequent annealing (400–600 °C).^35^ Additionally, several metal precursors can be added to the coating solution to generate metal-doped AT electrodes. Of particular interest, nickel-doped AT electrodes (NAT/Ni–Sb–SnO_2_) have been observed to coproduce O_3_ in addition to ^•^OH,^36^ and facilitate enhanced degradation of a number of organic pollutants when compared to AT electrodes.^37,38^ The fabrication method of both AT and NAT electrodes can facilitate the fabrication of high-surface-area flow-through electrodes,^31,39^ which can enhance mass transport and faradaic efficiency and therefore the electrochemical generation of ferrate.

In this study, the circumneutral electrosynthesis of ferrates was investigated using a heterojunction NAT and AT electrodes. To date, the circumneutral electrosynthesis of ferrates has yet to be yielded with any electrode other than BDD. In this study, we highlight the successful generation of ferrates, including the first report of the circumneutral electrosynthesis of powerful intermediate state ferrate(IV) and ferrate(V) species, using these MMO and BDD materials. Moreover, an in-depth mechanistic study yields the a novel reaction pathway to Fe(VI) from Fe(III) via electrolysis and ozone. The study also includes oxidation kinetics, oxidant stability, and speciation, as well as application for water treatment purposes, presenting a potentially powerful alternative to conventional and costly BDD electrodes and wet chemical synthesis processes for conventional ferrate(VI) generation.

## Materials and Methods

### Electrode Preparation

Three types of electrodes were used including (1) a single-layer AT-coated electrode on a Ti substrate, (2) a double-layer coated electrode consisting of a NAT top layer and an AT bottom layer, on a Ti substrate, and (3) a monocrystalline BDD electrode. The MMO electrodes were prepared using clean Ti plates (2 × 3 cm^2^), which were etched using a 1:4 HF:HNO_3_ solution for 1 min. The AT precursor solution was prepared using 360 mM SnCl_4_·5H_2_O (98%, Aldrich) and 40 mM SbCl_3_ (>99.0%, Aldrich). The NAT precursor solution was prepared using 360 mM SnCl_4_·5H_2_O, 15 mM SbCl_3_, and 4 mM Ni(OCOCH_3_)_2_·4H_2_O (98%, Aldrich). The respective metal oxide coatings were deposited on the Ti plates using a dip-coater (MTI Corporation Bridgman Crystal Growth Furnace), which included repeated intervals of dipping the substrate into the aqueous metal oxide precursor solution(s) for 20 s, drying at room temperature, followed by a calcination step by annealing at 600 °C for 10 min. This was repeated until a desired mass loading was achieved (AT electrode: 1.3 mg cm^–2^, NAT electrode: 1.3 mg cm^–2^ AT and 1.3 mg cm^–2^ NAT), whereby a final annealing step at 600 °C was performed for 1.5 h. The BDD electrode was commercially purchased from NeoCoat. It was prepared by a chemical vapor deposition process and had a thin-film (2–3 μm) monocrystalline layer on a 1 mm silicon substrate.

### Experimental Methods and Procedures

All experiments were performed using an undivided electrolysis cell (50 mL). All electrolyte solutions were continuously stirred with a magnetic stirrer at a rate of 400 rpm. The anode (AT, NAT and BDD) and cathode (stainless steel) were 6 cm^2^ for all experiments, separated by a 5 mm interelectrode gap. All tests were performed in a three-electrode configuration, using an Ag/AgCl reference electrode (BASI Inc.) and Biologic VSP-300 potentiostat. The base water matrix used for all experiments was a phosphate buffer (pH = 7.0, 0.1 M), composed of Milli-Q water, NaH_2_PO_4_, and Na_2_HPO_4_ (Millipore Sigma). The desired initial Fe^3+^ concentration was attained by addition of FeCl_3_ (Sigma-Aldrich). Current densities of 5, 10, and 15 mA cm^–2^ were investigated, to operate in a potential range avoiding excess oxygen evolution, while maximizing hydroxyl radical, DET and ozone formation, depending on each respective electrode. Samples for ferrate, probe or pollutant analysis were extracted from the electrochemical cell at a maximum volume of 0.5 mL throughout 60 or 90 min experiments. After electrolysis, ferrate solutions were centrifuged for 2 min at 5000 rcf and subsequently filtered using a 0.45 μm glass fiber syringe filter (Tisch) to remove any nonaqueous iron species (e.g., Fe^2+^/Fe^3+^ oxides and hydroxides).

Linear sweep voltammetry (LSV) and cyclic voltammetry (CV) experiments were also conducted under a scan rate of 50 mV s^–1^ in relevant electrolytes for electrode material characterization as well as oxidation mechanism and ferrate speciation analysis.

### Analytical Methods

Ferrate concentration was measured with an indirect spectrophotometric method using an ABTS (2,2′-azino-bis(3-ethylbenzothiazoline-6-sulfonic acid)) reagent (Sigma-Aldrich). In the presence of excess ABTS, ferrate(VI) oxidizes ABTS with a 1:1 M ratio, producing a light-absorbing radical cation (ABTS^•+^) with a visible UV-absorption maxima at 415 nm.^40−42^ Ferrate standards and samples were analyzed using a UV–vis spectrophotometer (Thermo Scientific Nanodrop 2000c) and a 1 cm quartz cuvette. Ferrate standards were prepared using potassium ferrate (99%) (Element 26) in a concentration range of 0–10 mM. Ferrate(VI) was also directly analyzed using direct UV–vis spectrophotometry at 530 nm in 1 and 5 cm quartz cuvettes, in a concentration range of 0–2 mM with co-occurring Fe^3+^ (dosed with FeCl_3_), to simulate water matrix conditions during electrolysis experiments and to understand the effects on UV-absorbance and ferrate(VI) stability. Raman spectroscopy (Renishaw inVia Qontor) and Fourier transform infrared spectroscopy (FTIR) (Thermo Scientific Nicolet iS50) were also pursued for a limited number of tests (operating methods are detailed in the Supporting Information). Free chlorine concentrations were also monitored using the DPD (*N*,*N*-diethyl-*p*-phenylenediamine) reagent (Hach DPD method 1012) and a DR 300 colorimeter.

Oxidant probe species and micropollutants, namely nitrobenzene (NB), carbamazepine (CBZ), and fluconazole (FCZ) (Sigma-Aldrich), were quantified using high performance liquid chromatography (HPLC), equipped with a ZORBAX Eclipse XDB-C18 column (Agilent, 2.1 × 50 mm^2^, 3.5 μm particles) and a UV detector at 254, 285, and 205 nm, respectively. The mobile phase, flowing at 0.5 mL min^–1^, was a composition of water with 0.1% formic acid and acetonitrile (ACN) under the gradient: 0 min, 10% ACN; 2 min, 10% ACN; 6 min, 95% ACN; 8 min, 95% ACN; 9 min, 10% ACN; 12 min, 10% ACN. An injection volume of 20 μL was used with a total runtime of 12 min for each sample.

## Results and Discussion

### Electrode Performance

Nonactive electrodes are characterized by their high oxygen evolution reaction (OER) overpotential.^43^ The NAT and AT electrodes have previously been shown to have oxygen evolution potential at ∼2.4 V_RHE_.^38^ BDD demonstrates even higher OER potential at ∼2.7 V_RHE_^44^ (see Figure S1 in the Supporting Information). One primary mechanism of oxidation, with respect to all three electrode materials (e.g., BDD, NAT, and AT), is mediated through the generation of ^•^OH, which was previously demonstrated by Zhang et al. through radical scavenging studies involving nitrobenzene and benzoic acid^38^ and widely reported for BDD electro-oxidation.^2^ Moreover, when Cl^–^ is present in the water matrix, all three electrodes are observed to generate reactive chlorines species (RCS). The NAT electrode is unique in its O_3_ production capacity. Equilibrium aqueous O_3_ concentration can reach as high as ∼4.7 mg L^–1^ in the absence of chloride and goes down with higher chloride concentration.^38^ The BDD electrode is the only material of the three that is shown to facilitate direct electron transfer (DET)-mediated oxidation. Specifically, DET is known to contribute to FCZ degradation during BDD electrolysis.^45^

### Ferrate Generation

Ferrate generation experiments were first performed using an initial FeCl_3_ concentration of 10 mM and a current density of 10 mA cm^–2^, with the NAT, AT, and BDD electrodes. Control experiments were run in parallel with 30 mM NaCl, yielding an equivalent Cl^–^ concentration, to understand the oxidative effect of cogenerated reactive chlorine species (RCS) during ferrate experiments. Additional control experiments were conducted to determine whether phosphate active species could also be generated, whereby electrolysis was performed in the presence of the base PBS water matrix and subsequently analyzed for any oxidative species.

For all anode materials, evidence of ferrate generation was observed using the ABTS quantification method^40,41^ (see Figures S2–S12 for all ABTS absorbance data). While the circumneutral generation of ferrate has been recently reported using BDD,^24^ this is the first evidence of ferrate generation using both NAT and AT electrodes. Faradaic efficiencies of 22.5, 21.3, and 12.0% were yielded for the BDD, NAT, and AT electrodes, respectively (see Figure 1b).

**Figure 1:**
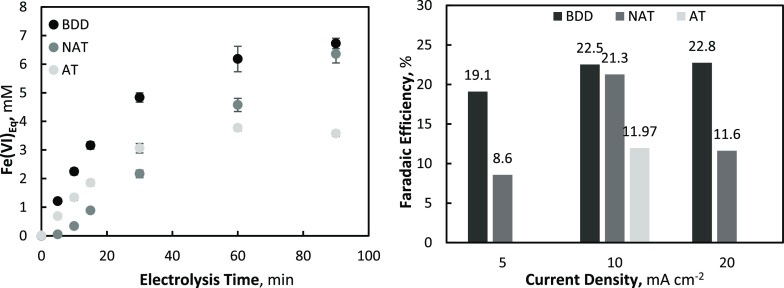
(A) Ferrate(IV/V/VI) generation (a) using BDD, NAT, and AT electrodes with a Fe^3+^_0_ = 10 mM and 10 mA cm^–2^. Fe(VI)_Eq_ = Equivalent oxidative capacity of Fe(VI) with ABTS (1:1 molar ratio). (B) Faradaic efficiency of ferrate (IV/V/VI) generation at 5, 10, and 15 mA cm^–2^ and with a Fe^3+^_0_ = 10 mM.

**Figure 2:**
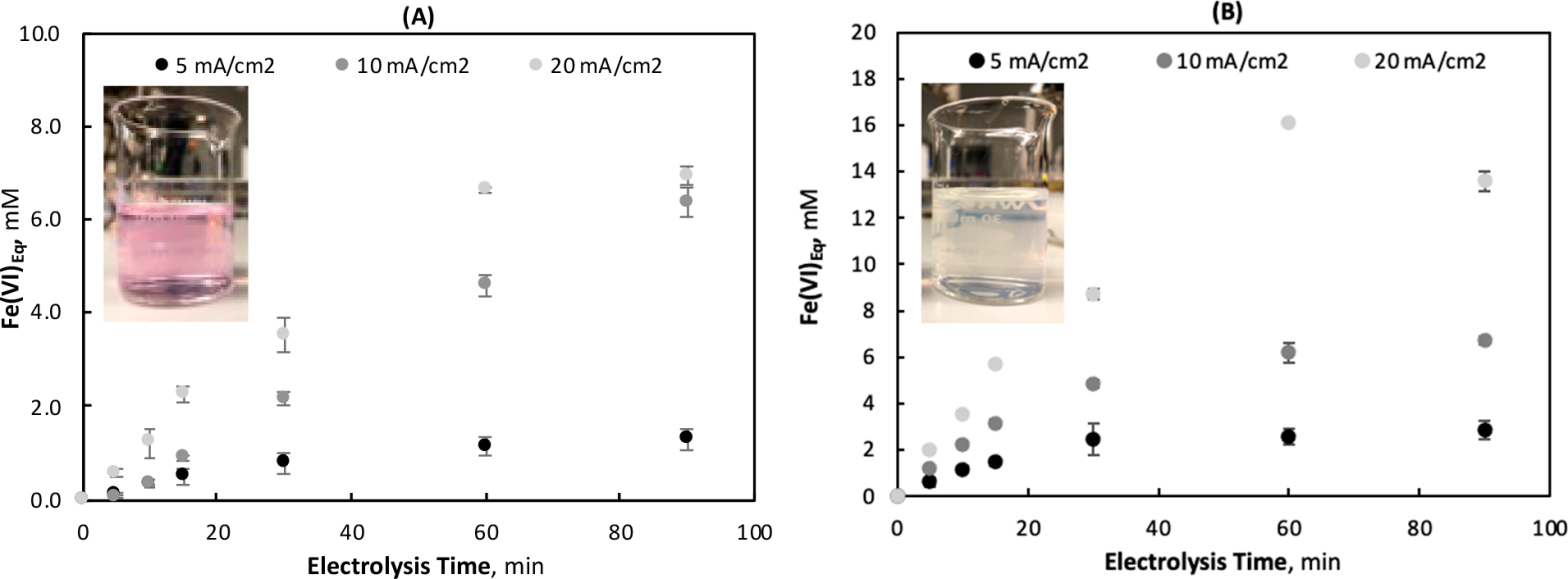
Ferrate(IV/V/VI) generation with a Fe^3+^_0_ = 10 mM at 5, 10, and 20 mA cm^–2^ using (A) NAT electrode and (B) BDD electrode. Inset figures are final ferrate solutions after 90 min of electrolysis. Fe(VI)_Eq_ = Equivalent oxidative capacity of Fe(VI) with ABTS (1:1 molar ratio).

Qualitatively, the final ferrate solutions were significantly different (see Figure 2 inset). While the NAT electrode produced a purplish/pink solution that is characteristic of ferrate(VI),^9^ both the BDD and AT electrodes produced a yellowish/white solution (see Figure 2), indicating that ferrate(VI) was not produced. The electrosynthesis of oxidative iron species was also observed to increase with both current density (20 mA cm^–2^) and initial Fe^3+^ concentration (15 mM) (see Figure S13 for all ABTS absorbance data). During NAT experiments, ferrate yields of 12.8, 63.5, and 69.4% were achieved during 5, 10, and 20 mA cm^–2^ operations. However, the Faradaic efficiency was maximized during 10 mA cm^–2^ electrolysis at 21.3%. Similarly, during BDD electrolysis, the greatest Faradaic efficiency was yielded during 10 mA cm^–2^ (see Figure 1b), with yields of 28.5, 67.2, and 135.8% during 5, 10, and 20 mA cm^–2^ operations. Average cell potentials of 3.5, 3.9, and 4.7 V were yielded during NAT electrosynthesis, while BDD potentials recorded were 4.7, 5.6, and 6.9 V during 5, 10, and 20 mA cm^–2^ operations, respectively. At 20 mA cm^–2^ BDD operations, as well as for all current density conditions using an initial FeCl_3_ concentration of 15 mM, ABTS results suggested that ferrate(VI) yields exceeded 100%, indicating errors associated with the ABTS method for ferrate(VI) quantification.

In theory, ferrate(VI) can facilitate the exchange of 3 mol of electrons for every 1 mol of ferrate(VI) through its subsequent conversion to Fe(V), Fe(IV), and Fe(III). In phosphate buffer, however, it has previously been found that ferrate(VI) has a 1:1.18 ratio with ABTS to form ABTS^•+^ in circumneutral pH, whereby the subsequent oxidative effect of Fe(V) and Fe(IV) are muted through rapid autodecomposition.^46^ Under the same conditions, roughly 9% of Fe(V) was found to react with ABTS to form Fe(IV), whereby 93% of that Fe(IV) subsequently reacting with ABTS to form ABTS^•+46^. Achieving ferrate yields greater than 100% using the ABTS method (which assumes a 1:1 reaction stoichiometry) for BDD electrosynthesis experiments suggests that Fe(V), or some combination of Fe(V) and Fe(IV), is formed, resulting in a 1:1–2 molar reaction ratio. The ABTS technique, and the resulting molar reaction ratio, has been previously used to identify the role of intermediate ferrate species (e.g., Fe(IV/V)) in water treatment processes.^47^

The generation and use of Fe(IV) and Fe(V) species, particularly for water treatment processes, has been a recent topic of much research interest and has primarily been reported through the activation of ferrate(VI).^47^ The activation of ferrate(VI), to exploit the high oxidation capabilities of Fe(IV/V), has been investigated using a number of strategies including the use of acid,^48^ UV,^49^ and metal cations like Fe(III),^50^ among many other organic and inorganic activators.^47^ Of particular relevance, the role of Fe(III) in Fe(IV/V) generation is notable due to its efficacy toward Fe(IV) generation and its co-occurrence during ferrate electrosynthesis.

### Ferrate Speciation

Several difficulties arise when conducting a speciation study on ferrate(IV/V/VI), particularly for direct detection and quantification of low concentration aqueous solutions. Both Raman spectroscopy and Fourier transform infrared (FTIR) spectroscopy were used to analyze the ferrate solutions obtained during BDD and NAT electrolysis, but no differentiating peaks were observed (see Supporting Information for Raman and FTIR spectra graphs, Figures S14 and S15, respectively). Moreover, high-valent iron-oxo complexes can exist in various forms and the exact structures are still largely unknown.^47^ For example, the synthesis of ferrate(IV), by hydroxyl radical-mediated oxidation of Fe(III), in phosphate, pyrophosphate, and carbonate solutions has been reported. The resulting ferrate(IV) species include one or more hydroxide, pyrophosphate, or carbonate ligand (L_m_). However, the exact Fe^IV^-L_m_ structure and number of ligands attached to the central iron atom are not known.^51,52^ It should also be noted that phosphate has previously been observed to function as a Fe(IV/V) ligand (e.g., Fe^V^-L_m_ and Fe^IV^-L_m_),^47^ which may play a crucial role in ferrate speciation in this study due to the presence of phosphate in the base PBS water matrix. The stability of ferrate species has also been observed to vary depending on exact structure and the ligand(s) coordinated to the iron atom.^52^

**Figure 3:**
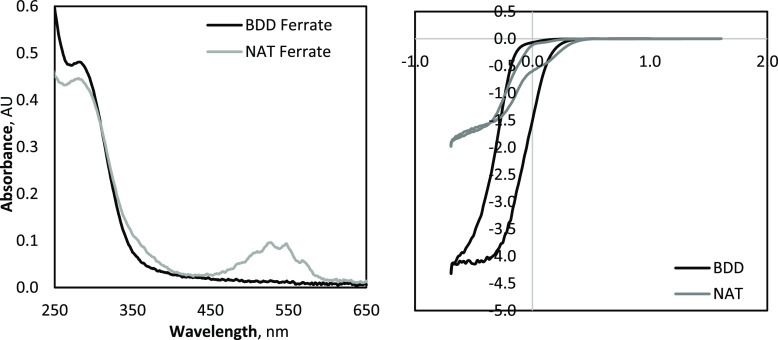
UV-spectra (left) and cyclic voltammograms (right) of final BDD and NAT ferrate solutions after 90 min of electrolysis at 10 mA cm^–2^ and an initial FeCl_3_ concentration of 10 mM (CV condition: scan rate 50 mV s^–1^, 0.0 V vs *E*_OC_ to −1.3 V vs ref.)

Direct colorimetry can also be used to identify ferrate(VI) in solution,^53,54^ at a much lower molar absorption coefficient than that of ABTS; therefore, higher concentrations and/or longer UV path lengths are required. Using a 5 cm quartz cuvette, both the BDD and NAT electrogenerated ferrates were analyzed (see Figure 3). The UV absorbance spectra confirmed the qualitative observations, whereby the solution produced with the NAT electrode showed evidence of ferrate(VI) with an absorbance maximum at 526 nm.^40^ This peak is slightly higher than that of ferrate(VI) observed with the chemical standard (using K_2_FeO_4_) and the peak shapes are notably different, as seen in Figure S35 with a maximum at 524 nm. When Fe(III) was added to the K_2_FeO_4_ mixture, however, the same absorbance maximum was achieved (see Figure S35). Moreover, while the NAT-produced ferrate(VI) solution was more pink (compared to a purple color traditionally associated with a high concentration of ferrate(VI)), it was similar to the color yielded using K_2_FeO_4_ and Fe(III). These results indicate that the lighter color observed during NAT electrolysis is due to either the PBS and/or co-occurring Fe(III) cations in solution. A secondary shoulder between 275 and 310 nm was observed for both the BDD and NAT derived ferrates, which is also characteristic of ferrate(VI) UV-spectra.^40^ No absorbance maxima was observed for the BDD solution, including at 530 nm (e.g., ferrate(VI) absorbance peak in the presence of Fe^3+^), conclusively indicating the absence of ferrate(VI). Cyclic voltammetry provided further direct evidence of two different iron species in the BDD and NAT solutions, as seen by the different reduction peaks in Figure 3.

To further understand ferrate speciation between the BDD and NAT derived solutions, the use of selective organic probes was pursued. The recalcitrance of nitrobenzene (NB) to ferrate(VI) oxidation has previously been reported.^15,55,56^ Using an initial concentration of 0.1 mM, no detectable NB degradation was observed to occur as a result of the NAT produced ferrate(VI), when compared to the control study containing RCS produced during electrolysis of the NaCl control solution (see Figures S16 and S17). During parallel experiments using the BDD-derived solution, NB degradation was observed to increase in the presence of ferrate(IV/V), when compared to the control solution containing only RCS, with pseudo first-order reaction rate constants of 0.0055 and 0.0047 min^–1^, respectively (see Figure S18).

Carbamazepine (CBZ) was also selected as a suitable probe, particularly in the water matrix postelectrolysis, which contains a high concentration of RCS, due to its relative persistence in highly chlorinated waters.^57,58^ With all electrodes (NAT, AT, and BDD), minimal CBZ degradation was observed during control (NaCl) experiments. Ferrate(VI), produced using the NAT electrode, facilitated the slow degradation of CBZ over ∼15 min of mixing, yielding a second-order reaction rate constant of 1.4 M^–1^ s^–1^. The degradation rate of CBZ with ferrate(VI) has been previously observed to be highly dependent on pH, with *k*_*2*_ constants ranging from 0.1 to 70 M^–1^ s^–1^ between pH conditions of 8 to 6, respectively,^59,60^ which is in agreement with the present study. Significantly faster CBZ oxidation was observed using the BDD-generated ferrate(IV/V), whereby 83% of CBZ degradation took place in the first 20 s of mixing, yielding an apparent first-order reaction rate constant of 0.08 s^–1^. This reaction rate is similar to that which has been observed for CBZ degradation with permanganate/Mn(VII)/MnO_4_^–^,^60^ an analogous highly oxidative manganese species also used for aqueous micropollutant degradation.^61−64^ The pseudo first-order reaction rate constant of permanganate with CBZ was found to increase with the initial permanganate concentration, whereby a *k′* ≈ 0.045 s^–1^ was yielded with an initial permanganate concentration of 160 μM. Although, in general, ferrate(VI) is more highly oxidative than the permanganate ion, the latter was reported to degrade CBZ more readily due to its high reactivity with olefin groups.^65,66^ At present, no previous studies have been published investigating the used of ferrate(V) or ferrate(IV) on the degradation of CBZ, but is observed to perform similarly to permanganate. CBZ degradation was also observed using the ferrate(IV/V) generated using the AT electrode. Similar to the BDD derived ferrate solution, rapid degradation was observed in the first 20 s (see Figure S22), with an absolute CBZ removal much less than BDD due to the significantly lower initial concentration of ferrate(IV/V).

Fluconazole (FCZ) was used as a probe, as it is recalcitrant to both chlorine and chlorine radicals (Cl_2_^•–^). Similar to CBZ, no FCZ degradation was observed during control experiments with both the NAT and BDD producing ferrate solutions. Moreover, ferrate(VI), synthesized using the NAT electrode, did not yield any detectable oxidation of FCZ. A small amount of FCZ degradation was observed using the ferrate(IV/V), produced with the BDD electrode, once again highlighting the greater oxidation potential of these iron species compared to ferrate(VI). In agreement with previous researchers, the BDD produced ferrate(IV/V) consistently yielded greater degradation rates when compared to ferrate(VI) for all organic micropollutant tested.^16,17^ A complete set of the CBZ and FCZ degradation data can be found in the Supporting Information (Figures S19–S24).

**Figure 4:**
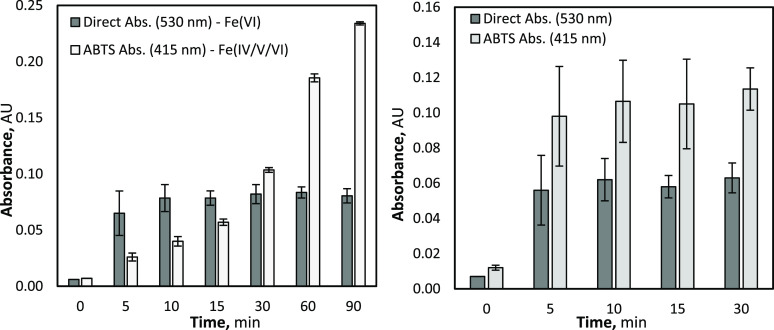
ABTS (415 nm) and direct (530 nm) UV–vis spectrophotometric results from NAT electrolysis at 10 mA cm^–2^ and FeCl_3_ of 15 mM (left), and O_3_ oxidation with an initial FeCl_3_ of 15 mM (right).

To better understand the ferrate speciation during NAT electrolysis, a combination of ABTS and direct UV-spectrophotometer analysis was conducted in parallel at 415 and 530 nm to evaluate the relative ferrate(IV/V) and ferrate(VI) synthesis, respectively. The absorbance related to the generation of ferrate(VI) increased over the initial 10 min of electrolysis; thereafter, it stabilized for the remainder of the experiment. The ABTS absorbance, which reflects oxidation by ferrate(IV), ferrate(V), and ferrate(VI), continued to increased significantly throughout the entirety of electrolysis (see Figure 4). These results indicate that only a small fraction of the initial Fe^3+^ is converted to ferrate(VI), 1.65 mM (±0.2 mM) under these conditions (10 mA cm^–2^ and [Fe^3+^]_0_ = 15 mM), in a fast reaction within 10 min of starting electrolysis, and remained constant thereafter. Although ferrate(VI) generation reaches a plateau, the formation of ferrate(IV/V) continues to be facilitated throughout the 90 min of electrolysis.

Each of the techniques used in this study, however, is indirect analysis for ferrate speciation. In future studies, it would be important to incorporate direct analysis, particularly the use of Mössbauer spectroscopy. In order to use Mössbauer spectroscopy, reactor design and current efficiency limitations of the current setup would need to be improved, as a higher concentration of ferrates in a solid sample would be required.^47^

### Ferrate Stability

A long-term stability study was performed on both the ferrate(VI) (e.g., NAT produced ferrate) and ferrate(IV/V) (e.g., BDD produced ferrates) to understand their relative self-decomposition in ambient temperature (20 °C) and light conditions. Similar to the previously described ferrate generation and speciation tests, all stability tests were performed in parallel with control experiments using NaCl solutions and monitoring the degradation of cogenerated RCS. Using the same initial FeCl_3_ (15 mM) concentration with both the NAT and BDD electrodes, after 60 min of electrolysis, initial Fe(VI)_Eq_ concentrations of 3.9 and 13.6 mM were achieved after centrifugation and filtration, respectively. The ABTS absorbances were subsequently monitored for the ferrate and RCS control solutions over 70 days.

The oxidant species stability of all solutions (e.g., ferrate and control) degraded similarly; however, some outlier ABTS absorbances were observed between days 3–11 for the BDD ferrate(IV/V). Unlike the remainder of the stability data, these outliers included large variations in ferrate(IV/V) concentrations, and no conclusive evidence had been gathered to explain these outliers. One possible explanation may be related to the decomposition of Fe(V) to Fe(IV) and/or Fe(III) during sampling by oxidation with nonaqueous iron hydr(oxides) (e.g., reduced ferrate products). All ABTS stability data are included in the Supporting Information (Figures S25–S28).

**Figure 5:**
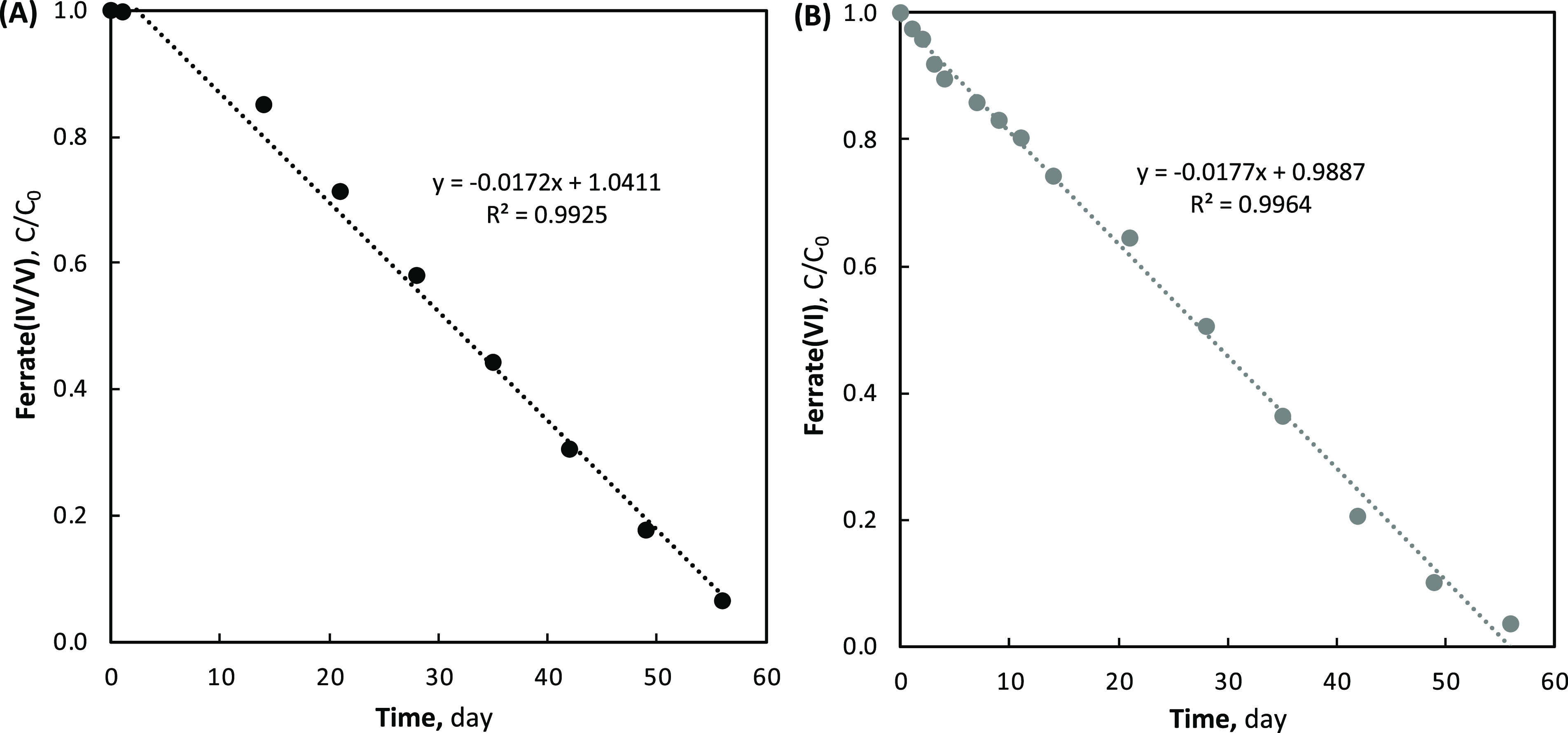
Normalized ferrate degradation with (A) BDD derived ferrate(IV/V) (Fe(VI)_Eq_ = 13.6 mM) and (B) NAT derived ferrate(VI) (Fe(VI) = 3.9 mM).

When accounting for the effect of RCS, zero-order degradation was observed for both the NAT produced ferrate(VI) and BDD produced ferrate(IV/V). Excluding the previously describe unaccountable outlier samples, high coefficients of determination were yielded for both ferrate solutions, with degradation rate constants of 0.0691 and 0.234 mM day^–1^ for ferrate(VI) and ferrate(IV/V), respectively. However, when degradation rates were normalized for initial ferrate concentrations differences, both solutions were observed to degrade at a very similar rate (see Figure 5).

CVs were also run each week throughout the duration of the stability tests, with a particular interest in determining whether multiple peaks would develop/regress in the BDD solution, to provide insights toward Fe(V) and Fe(IV) species. Only a single reduction peak at ∼−0.3 V_RHE_ is visible in the ferrate(IV/V) CVs throughout the 6 weeks of the degradation study (see Figure 3). Interestingly, two reductions peaks are visible for the NAT produced ferrate(VI) throughout the stability test, which may be associated with the reduction of Fe(VI) and subsequent reduction of Fe(IV/V). All CVs for both the NAT and BDD produced ferrates can be found in the Supporting Information (Figures S29 and S30).

### Ferrate Generation Mechanism

There are few differences between the predominant oxidation mechanisms, as it relates to BDD, NAT, and AT electro-oxidation. In the former, DET can be facilitated as a secondary pathway to the predominant ^•^OH-mediated oxidation. In the case of NAT electro-oxidation, appreciable amounts of O_3_ are generated, but it is also secondary to the predominant ^•^OH-mediated oxidation pathway.^38^ Although DET is exclusive to the BDD, it cannot be assumed to be the responsible mechanism for the electrosynthesis of ferrate(IV/V), as the AT electrode, which solely proceeds via ^•^OH-mediated electro-oxidation, also produces the same ferrate species as that of BDD. Therefore, it was hypothesized that O_3_ play a crucial role in facilitating the synthesis of ferrate(VI).

Three tests were performed to understand the role of O_3_ for the generation of ferrate(VI). First, after electrolysis and ferrate(IV/V) generation using the BDD electrode, O_3_ was purged into the solution and mixed over 30 min. In the second experiment, O_3_ was directly purged into the electrochemical cell during BDD electrolysis. The third experiment was similar to the first; however, the ferrate(IV/V) solution generation after BDD electrolysis was centrifuged and filtered to remove any reduced ferrate or hydrolyzed Fe^3+^. In all experiments, ferrates were analyzed using the colorimetric ABTS and direct method as well as through qualitative observations on the resulting solutions’ color.

**Figure 6:**
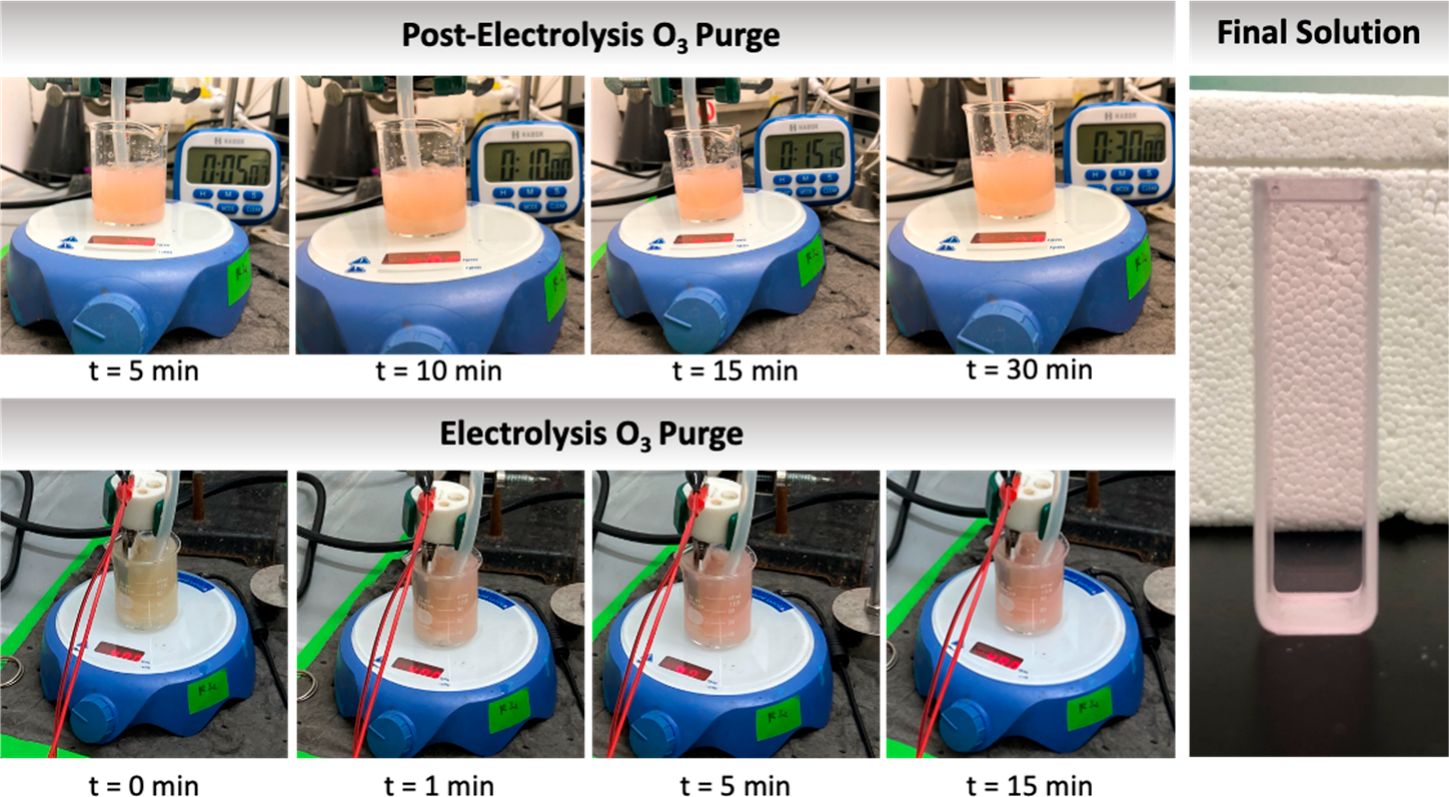
Ferrate(IV/V) oxidation experiments with O_3_ post and during electrolysis to form ferrate(VI).

For the first two experiments, ferrate(VI) was observed to form. In the first process, where O_3_ was purged into a mixing solution of ferrate(IV/V) post BDD electrolysis, the solution began to immediately turn from a yellowish/white color (see inset of Figure 2b) to the characteristic purple/pink solution that was yielded during NAT electrosynthesis experiments (see Figure 6). As the solution turned pink over the 30 min of O_3_ purging and mixing, indicating the generation of ferrate(VI), the ABTS absorbance decreased significantly, which is consistent with the ABTS data yielded during ferrate generation experiments with the NAT electrode. Parallel control experiments were also conducted to account for ABTS^•+^ formation due to O_3_ oxidation. ABTS absorbance data for both ferrate and control experiments can be found in the Supporting Information (Figures S31, S33, and S34).

A similar trend was observed when O_3_ was purged during electrolysis, whereby a pink/purple solution was yielded within minutes of current application (see Figure 6). The ABTS data suggested that appreciable amounts of Fe(IV/V) were still being generated; however, the absorbance was significantly greater than that yielded during NAT electrolysis but still greatly below the absorbances observed during the standard BDD ferrate generation experiments (i.e., without purging O_3_). These results highlight the relatively high current efficiency for BDD-iron reactions and reveal a potentially high efficiency reaction path for ferrate(VI) electro-generation in concurrence with O_3_ oxidation.

Lastly, during the third experiment where O_3_ was purged into a centrifuged and filtered solution of ferrate(IV/V) post BDD electrolysis, no evidence of ferrate(VI) generation was observed. These results suggested that ferrate(VI) synthesis was predominantly facilitated by the oxidation of Fe^3+^ by ozone rather than ferrate(IV/V) species by ozone. To further confirm these findings, ozone was purged directly into a Fe^3+^ solution ([FeCl_3_]_0_ = 15 mM) and the ABTS and direct UV-absorbance was measured to quantify the ferrate(IV/V) and ferrate(VI) generation, respectively. Control experiments were conducted with the ozonation a Cl^–^ containing solution ([NaCl]_0_ = 45 mM) and the base phosphate buffer solution to account for the effect of ABTS oxidation by RCS and/or ozone (control experiment data can be found in Figure S33). Over 30 min of purging and mixing, ferrate(VI) was observed to be generated, demonstrating the first evidence of an ozone synthesis pathway. Based on the control experiments, as well as previous research that found ozone to have a small reaction rate coefficient with Cl^–^ to form RCS,^67^ the primary mechanism for ferrate(VI) generation was determined to be by ozone oxidation. ABTS and direct UV-absorbance increased proportionately over the time of ozone purging, as seen in Figure 4. Although the purge-rate for ozone during these tests (0.011 L min^–1^) was much greater than that which occurs during NAT electrolysis (<1.8 × 10^–06^ L min^–1^ at 10 mA cm^–2^), more ferrate(VI) was produced during the latter process, suggesting some electrocatalytic effects of the NAT material, and/or to the coproduction of hydroxyl radicals and ferrate(IV/V) during electrolysis. This effect, however, is currently out of the scope of this project.

This study demonstrates that MMO materials, particularly NAT and AT electrodes, are capable of generating high oxidation state iron species ferrate(VI) and ferrate(IV/V). Moreover, this work provides the first extensive evidence of ferrate(IV/V) electrochemical generation in circumneutral pH conditions using the BDD, NAT, and AT electrodes. These intermediate state ferrate species were shown to be more highly oxidative against persistent organic pollutants, which are recalcitrant toward ferrate(VI) oxidation. Finally, a key mechanism for ferrate(VI) electrosynthesis using the NAT electrode was proposed, which was identified to be attributed to co-occurring ozone oxidation of Fe^3+^, demonstrating two novel reaction pathways for ferrate(VI) generation.

## Supporting Information Available

Cyclic voltammograms of BDD; ferrate ABTS calibration curves; ABTS absorbances during BDD, NAT and AT, ferrate, and control experiments; Raman and FTIR spectra of final BDD and NAT ferrate solutions; nitrobenzene, CBZ, and FCZ degradation using BDD and NAT derived ferrate and control solutions; ABTS absorbances and cyclic voltammograms for ferrate stability tests; ABTS absorbances of O_3_ purging experiments; UV-absorption spectra of ferrate(VI) from K_2_FeO_4_, K_2_FeO_4_, and Fe(III) and NAT-generated ferrate(VI) (PDF)
