# Supplementary material for: Novel Synthesis Pathways for Highly Oxidative Iron Species: Generation, Stability, and Treatment Applications of Ferrate(IV/V/VI)
Source: Environ Sci Technol. 2023 Feb 16;57(47):18700–9. doi: 10.1021/acs.est.2c09237 (PMC10690715; doi:10.1021/acs.est.2c09237)
Supplement: Supplementary file 1 — es2c09237_si_001.pdf [file es2c09237_si_001.pdf]

Supporting Information

Novel synthesis pathways for highly oxidative iron species: Generation, stability, and treatment applications of ferrate(IV/V/VI)

Sean T. McBeath,[#*] Yi Zhang,[#] Michael R. Hoffmann

[#]Co-First Authors

Linde Laboratories, California Institute of Technology, Pasadena CA 91125, United States

[#]S.T.M. and Y.Z. contributed equally to this paper.

[*]Corresponding Author: smcbeath@umass.edu

Tables: 0

Figures: 35

Total Pages: 19

S.1. Electrode Characterization

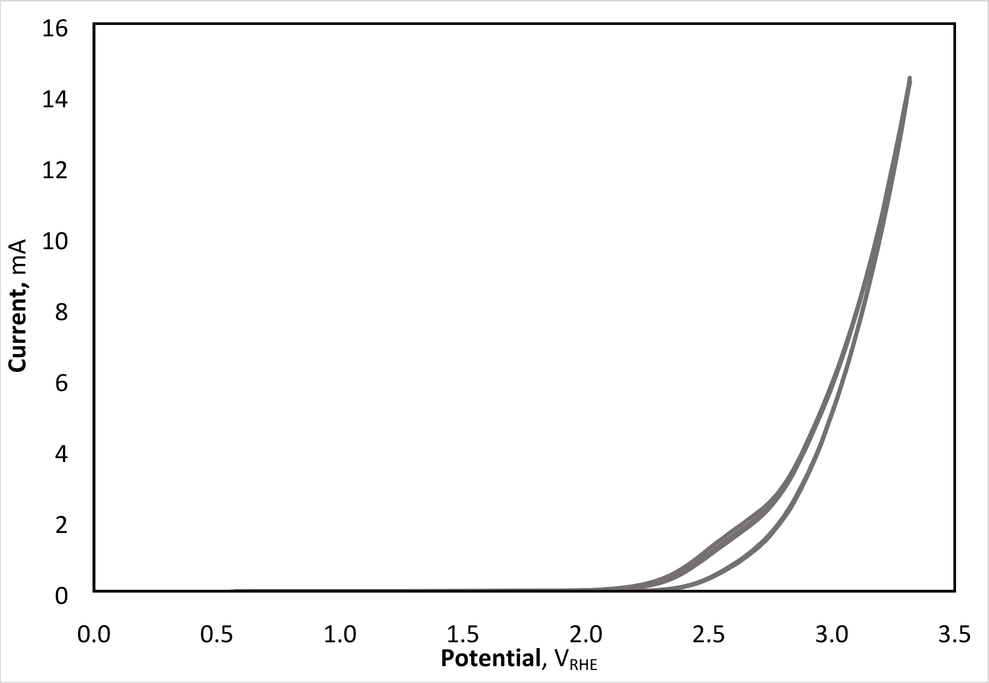

Figure S1. Cyclic voltammograms of BDD with OER potential at ~2.7 V_{RHE}. CV condition: scan rate 50 mV s^{-1}, 0.0 V vs. E_{OC} to +3.0 V vs. ref.

S.2. Ferrate Generation

The ABTS indirect ferrate quantification method was chosen due its sensitivity in determining low concentrations of Fe(VI). In a 50 mL erlenmyer flask, 5 mL of acetate buffer (pH=4.1), 1 mL of ABTS reagent (1 g/L) and 9 mL of MilliQ water is added. New ABTS reagent solutions were made prior to each electrolysis experiment and stored in the refrigerator at 4˚C to avoid degradation due to increased temperature and light exposure. A 0.5 mL ferrate sample was added to the Erlenmeyer flask, followed by 9.5 mL of phosphate buffer, to reach a final volume of 25 mL. This ABTS solution was then analyzed using the spectrophotometer at 415 nm, a visible maxima for $ABTS^{\bullet+}$. Ferrate concentrations were determined from experimental measurements as follows:

$$[Fe(VI)] = \frac{\Delta A_{415} V_f}{\varepsilon l V_s} \quad (1)$$

Where ΔA_{415}, V_f, $\mathcal{E}$, l and V_s represent the UV-absorbance at 415 nm, the final sample volume, the absorption coefficient as determined by the standards, the cell path length, and the volume of the ferrate sample.

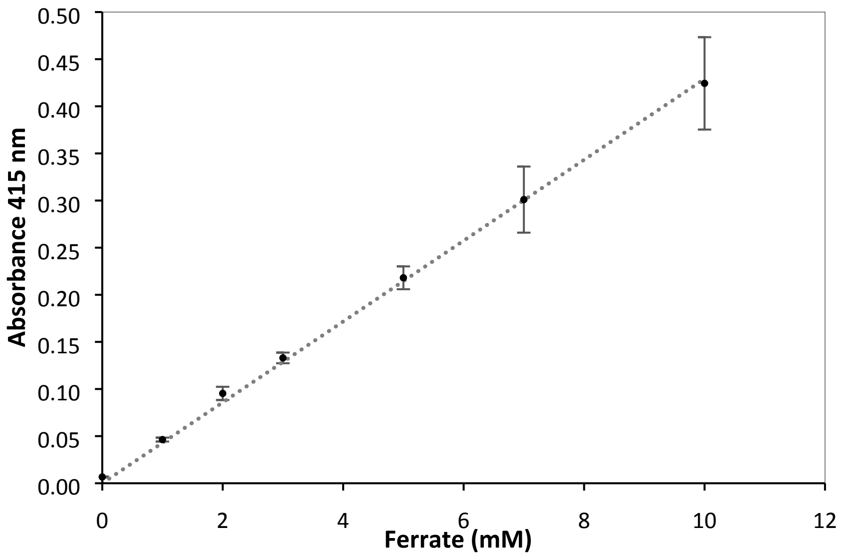

Figure S2. Ferrate(VI) ABTS calibration curves using K_2SO_4 (Element 26) in phosphate buffer (pH = 7.0), yielding a molar absorption coefficient of 0.043 mM^{-1} cm^{-1}.

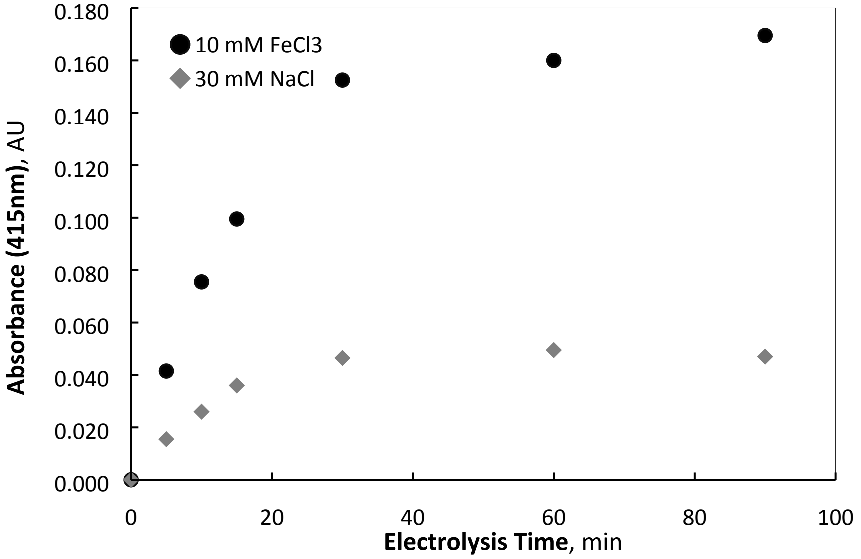

Figure S3. ABTS absorbance during BDD ferrate (5 mA cm^2, 10 mM $FeCl_3$) and control (5 mA cm^2, 30 mM NaCl) experiments.

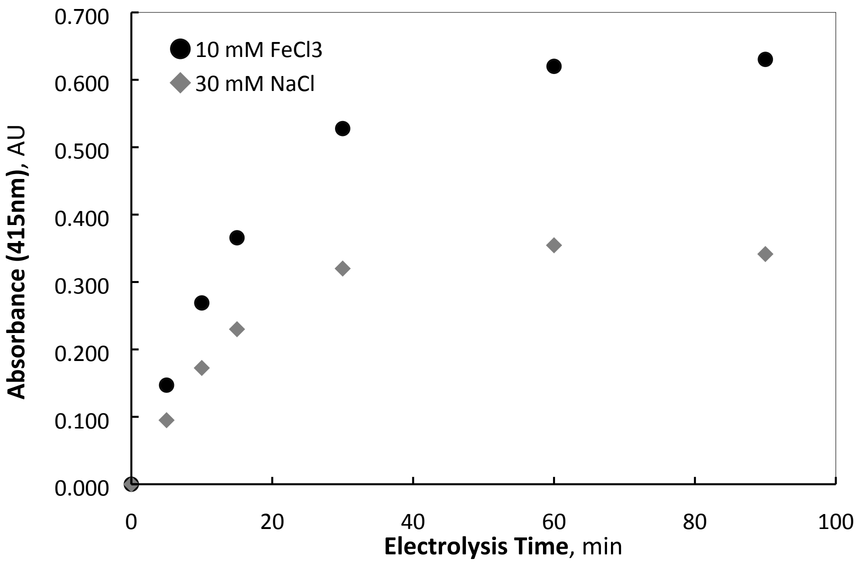

Figure S4. ABTS absorbance during BDD ferrate (10 mA cm[2], 10 mM $FeCl_3$) and control (10 mA cm[2], 30 mM NaCl) experiments.

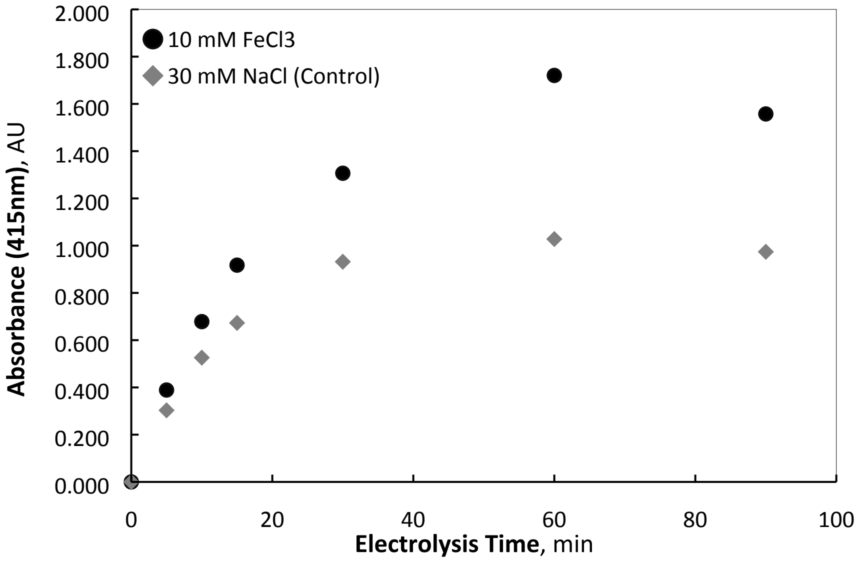

Figure S5. ABTS absorbance during BDD ferrate (20 mA cm[2], 10 mM $FeCl_3$) and control (20 mA cm[2], 30 mM NaCl) experiments.

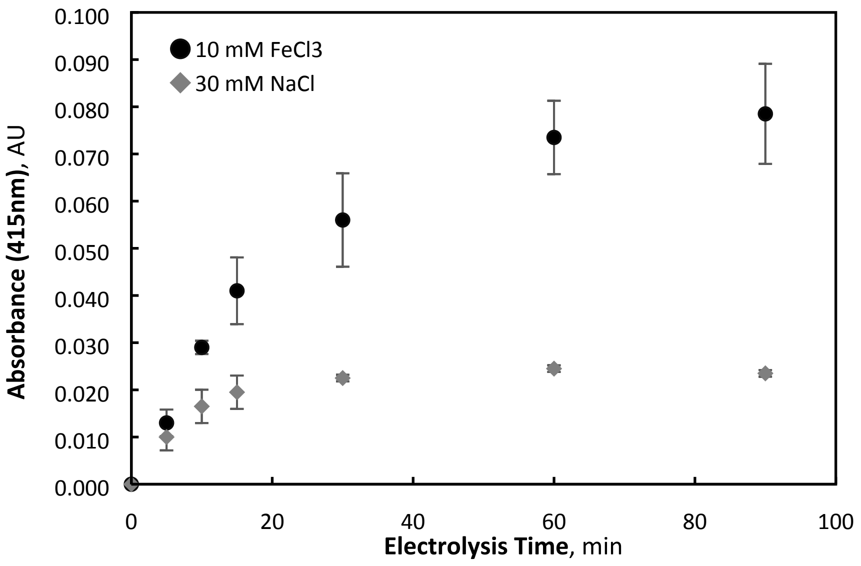

Figure S6. ABTS absorbance during NAT ferrate (5 mA cm^2, 10 mM $FeCl_3$) and control (5 mA cm^2, 30 mM NaCl) experiments.

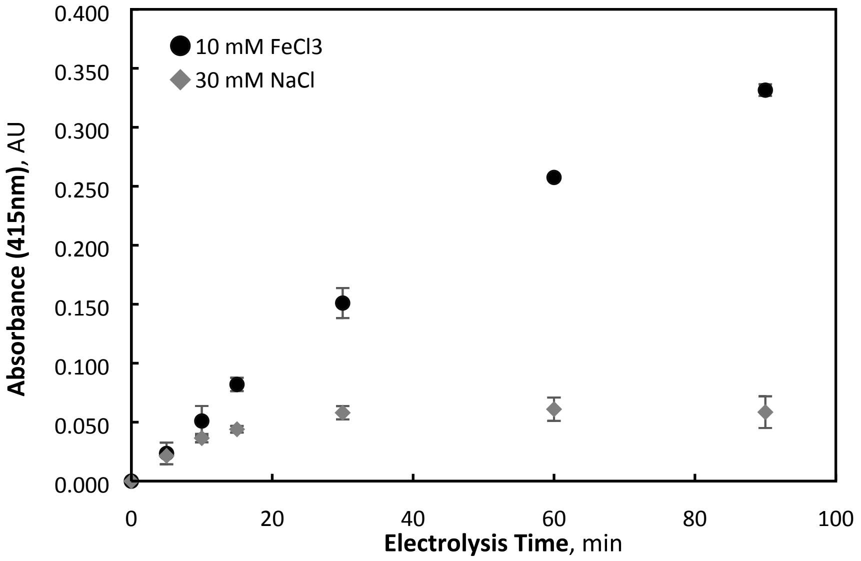

Figure S7. ABTS absorbance during NAT ferrate (10 mA cm^2, 10 mM $FeCl_3$) and control (10 mA cm^2, 30 mM NaCl) experiments.

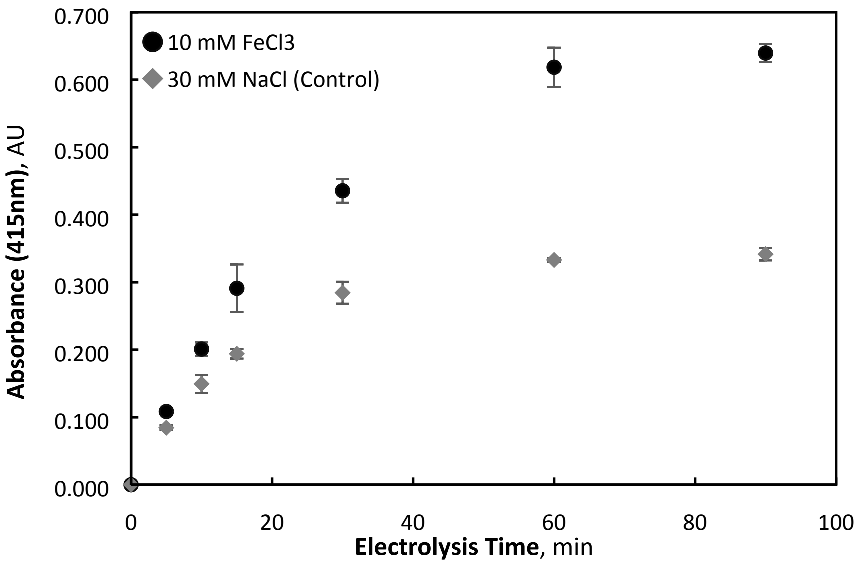

Figure S8. ABTS absorbance during NAT ferrate (20 mA cm^2, 10 mM $FeCl_3$) and control (20 mA cm^2, 30 mM NaCl) experiments.

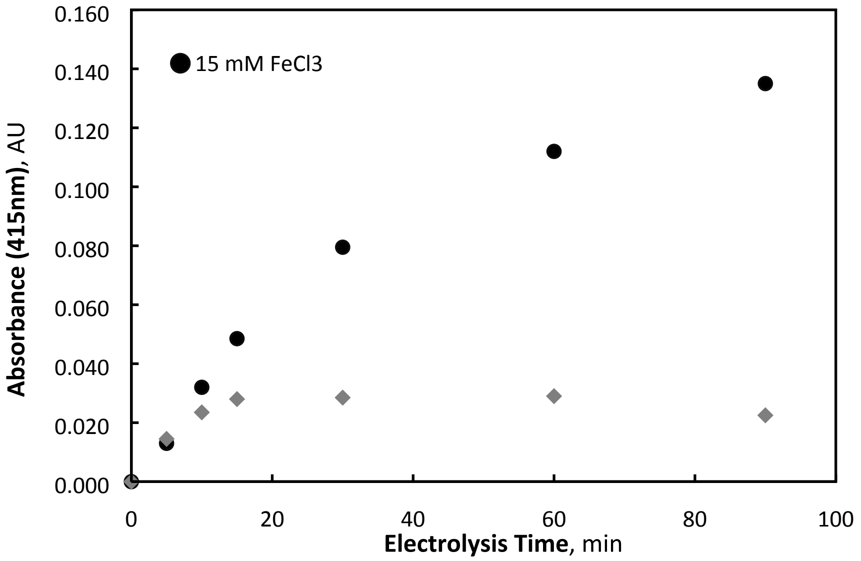

Figure S9. ABTS absorbance during NAT ferrate (5 mA cm^2, 15 mM $FeCl_3$) and control (5 mA cm^2, 45 mM NaCl) experiments.

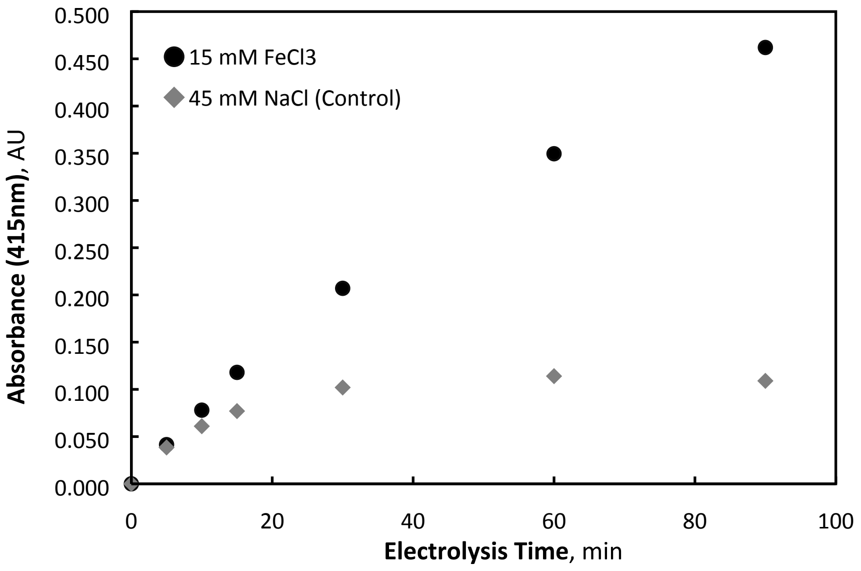

Figure S10. ABTS absorbance during NAT ferrate (10 mA cm^2, 15 mM $FeCl_3$) and control (10 mA cm^2, 45 mM NaCl) experiments.

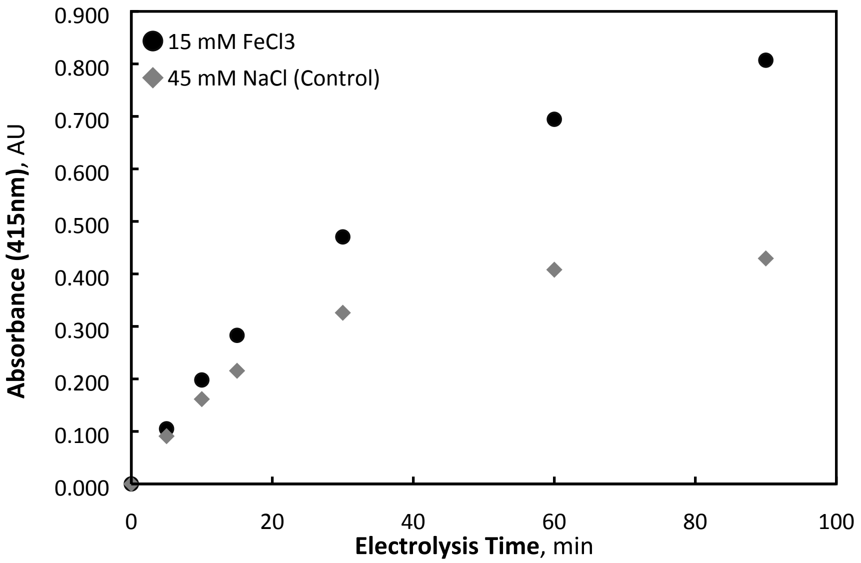

Figure S11. ABTS absorbance during NAT ferrate (20 mA cm^2, 15 mM $FeCl_3$) and control (20 mA cm^2, 45 mM NaCl) experiments.

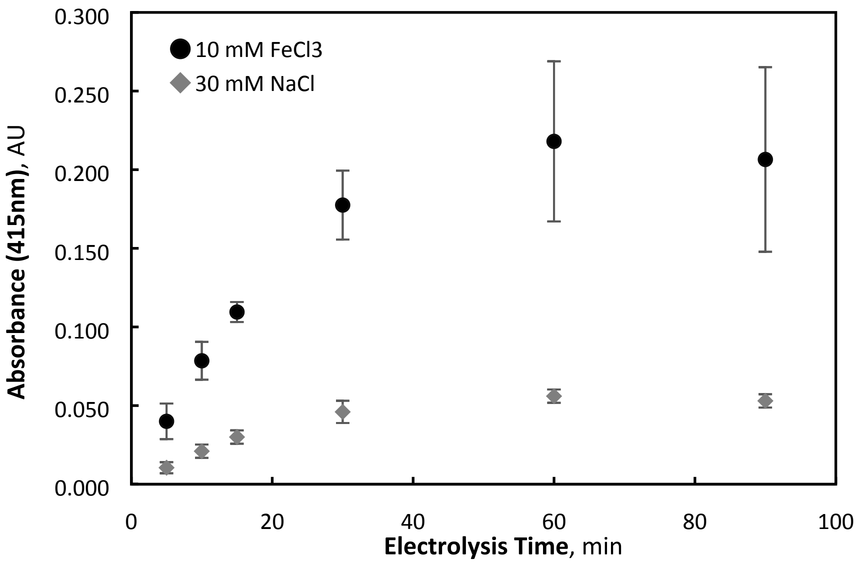

Figure S12. ABTS absorbance during AT ferrate (10 mA cm^2, 10 mM $FeCl_3$) and control (10 mA cm^2, 30 mM NaCl) experiments.

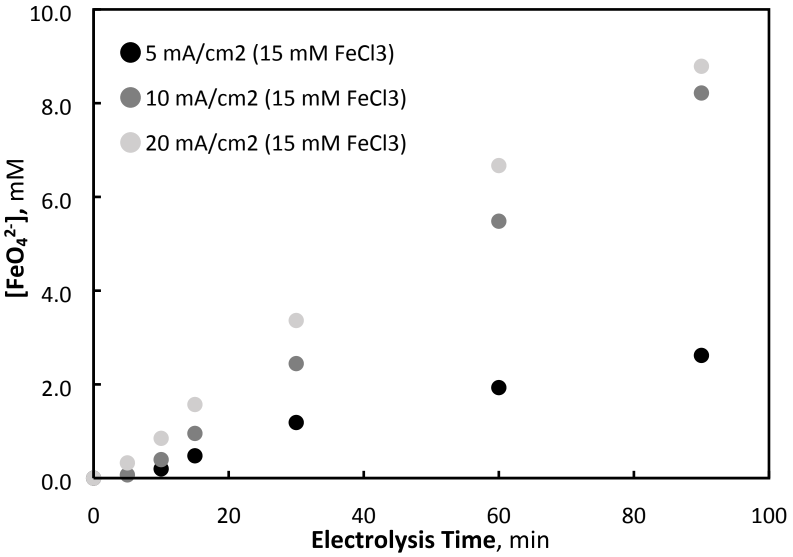

Figure S13. Ferrate(VI) generation using an NAT electrodes with a $Fe^{3+}{}_0$ = 15 mM and current densities of at 5, 10 and 20 mA cm^{-2}.

S.3. Ferrate Speciation

The BDD and NAT produced ferrate(IV/V/VI) solutions were analysed using Raman spectroscopy (Renishaw inVia Qontor) to determine whether the different ferrate species (e.g., Fe(IV), Fe(V) and/or Fe(VI)) could identified. Raman micro-spectroscopy was performed using a 514 nm laser as the exciting light. Calibration was done using a silicon wafer with a Raman band centered at 520.5 cm^{-1}.

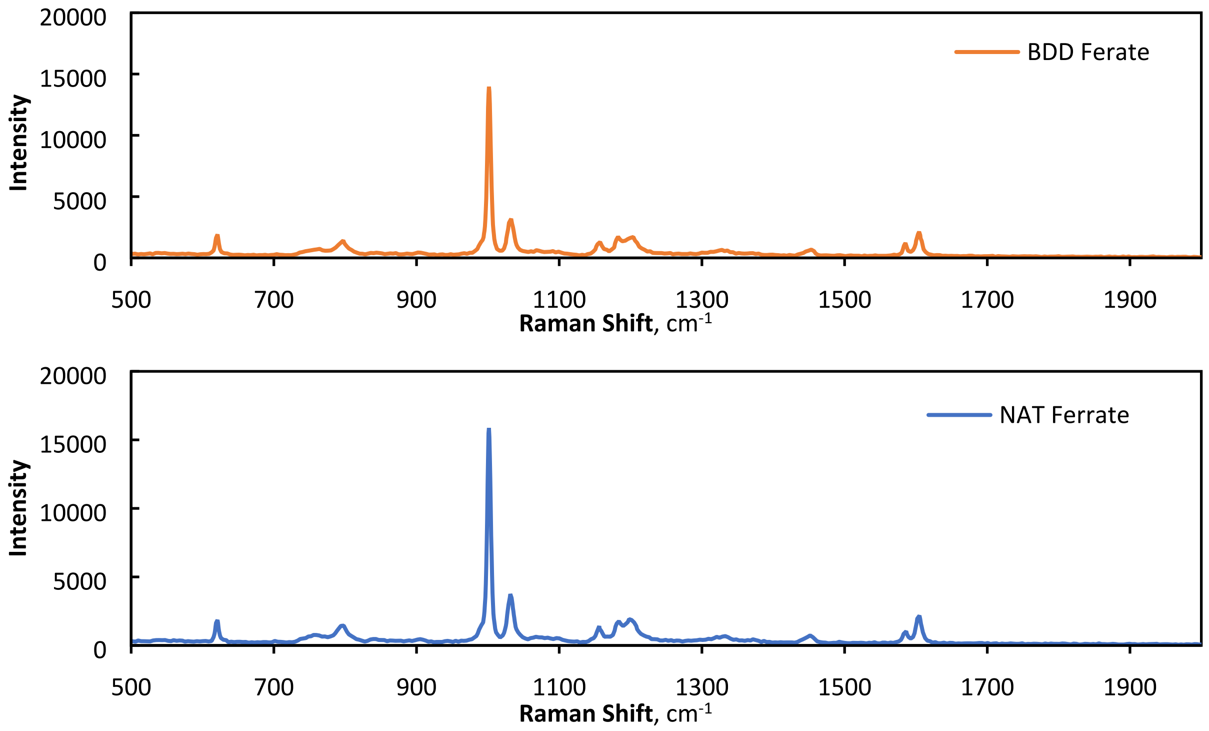

Figure S14. Raman spectra graph of final BDD and NAT ferrate solutions after 90 min of electrolysis at 10 mA cm^{-2} and an initial $FeCl_3$ concentration of 10 mM.

The BDD and NAT produced ferrate(IV/V/VI) solutions were also analysed using FTIR spectroscopy (Thermo Scientific Nicolet iS50) to determine whether the different ferrate species (e.g., Fe(IV), Fe(V) and/or Fe(VI)) could be identified.

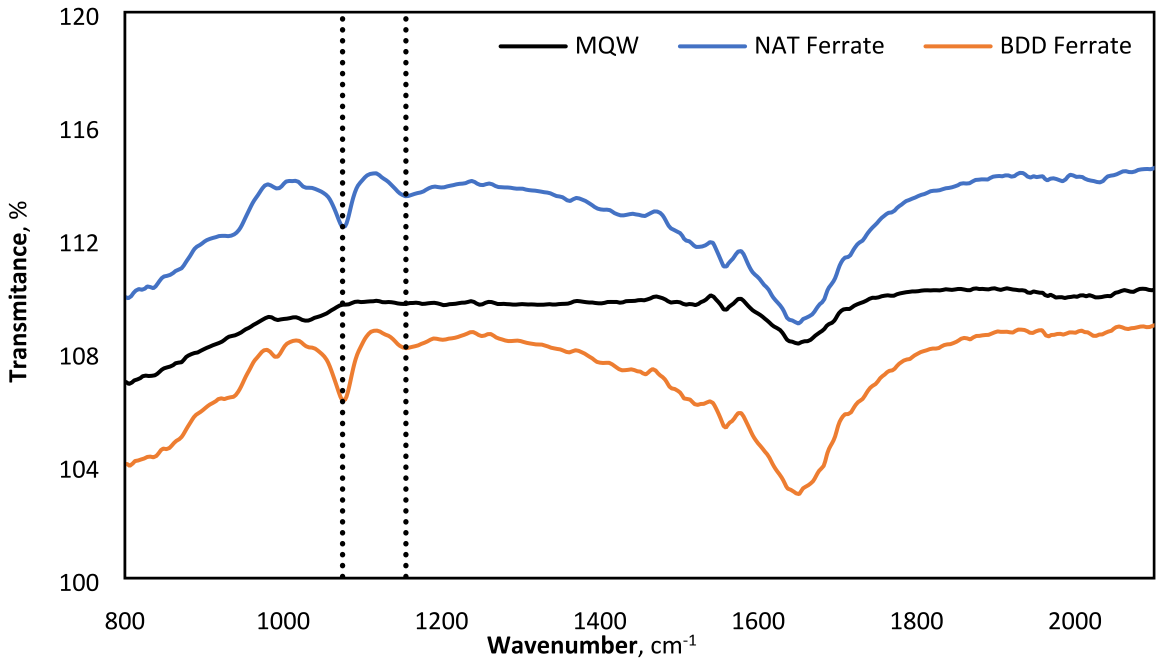

Figure S15. FTIR spectra graph of final BDD and NAT ferrate solutions after 90 min of electrolysis at 10 mA cm^{-2} and an initial $FeCl_3$ concentration of 10 mM.

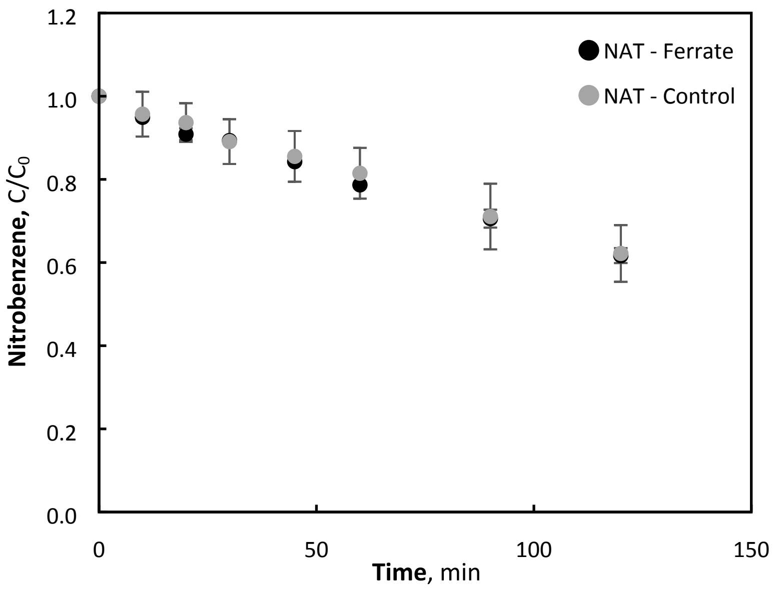

Figure S16. Nitrobenzene degradation using NAT derived ferrate(VI) and NaCl control solutions (NB_0 = 0.1 mM, Fe(VI) = 1.52 mM)

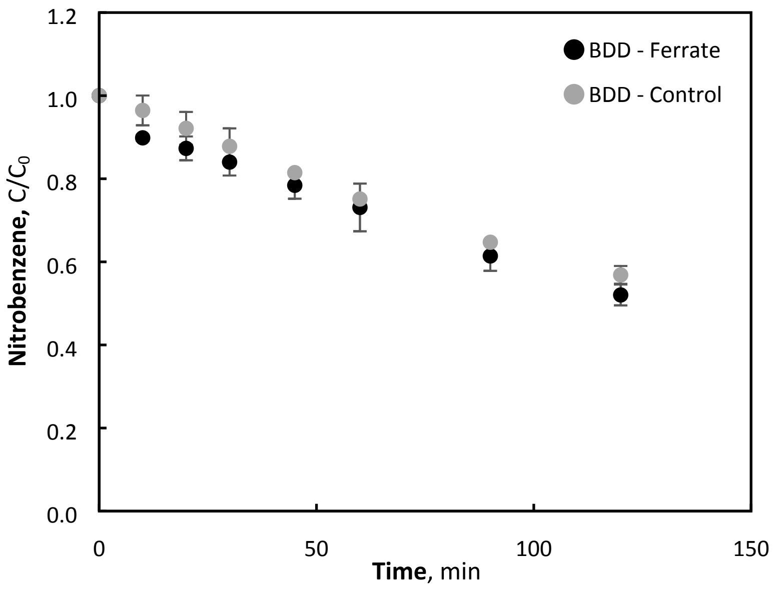

Figure S17. Nitrobenzene degradation using BDD derived ferrate(IV/V) and NaCl control solutions (NB_0 = 0.1 mM, $Fe(VI)_{Eq}$ = 3.83 mM)

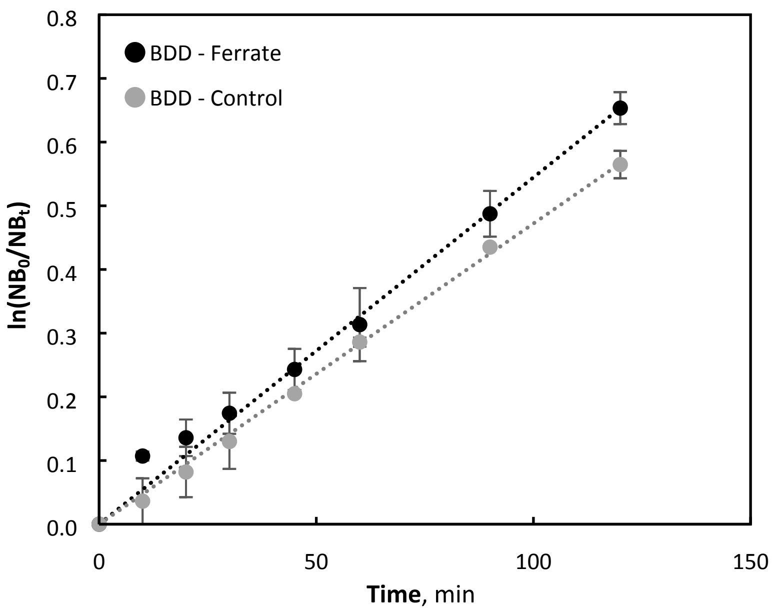

Figure S18. Nitrobenzene degradation using BDD derived ferrate(IV/V) and NaCl control solutions (NB_0 = 0.1 mM, $Fe(VI)_{Eq}$ = 3.83 mM)

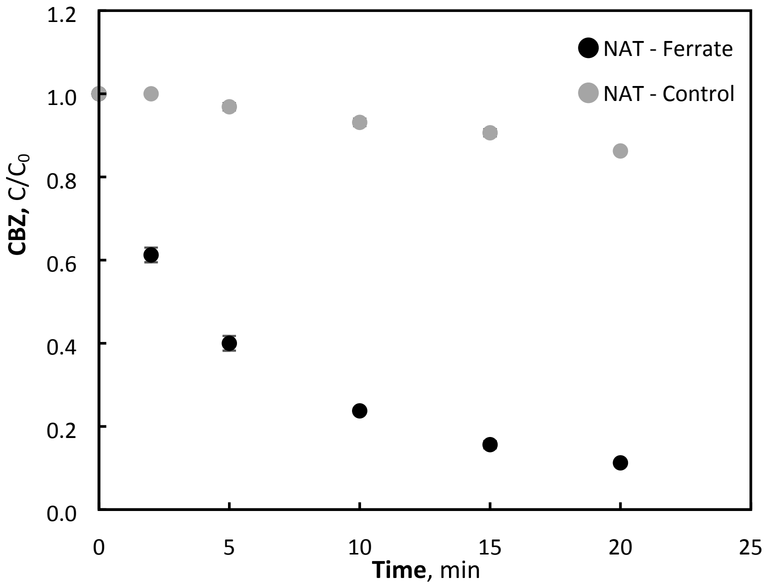

Figure S19. CBZ degradation using NAT derived ferrate(VI) and NaCl control solutions (CBZ_0 = 10 μM).

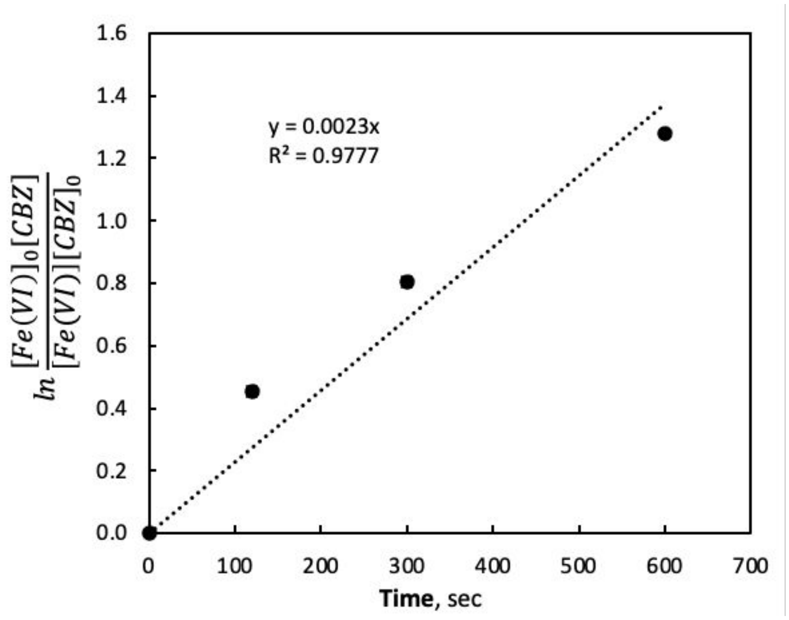

Figure S20. Second-order reaction rate determination of CBZ and NAT derived ferrate(VI) (CBZ_0 = 10 μM, $Fe(VI)_0$ = 1.66 mM).

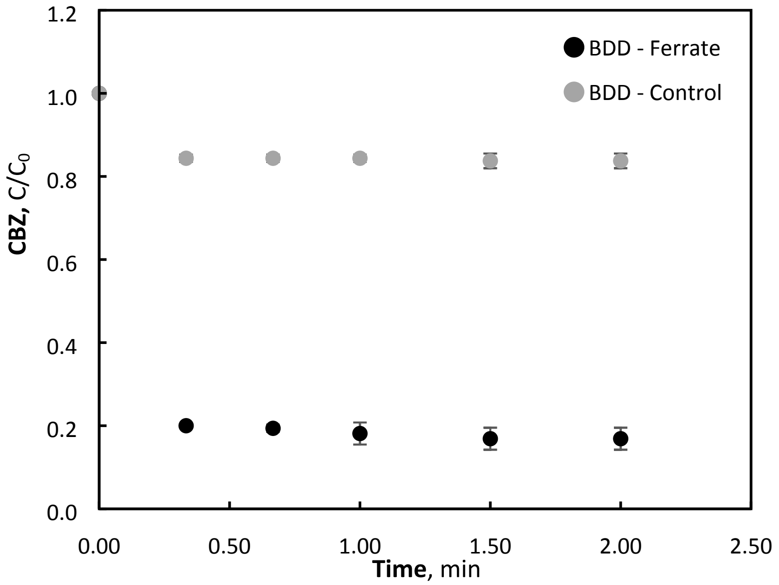

Figure S21. CBZ degradation using BDD derived ferrate(IV/V) and NaCl control solutions (CBZ_0 = 10 μM).

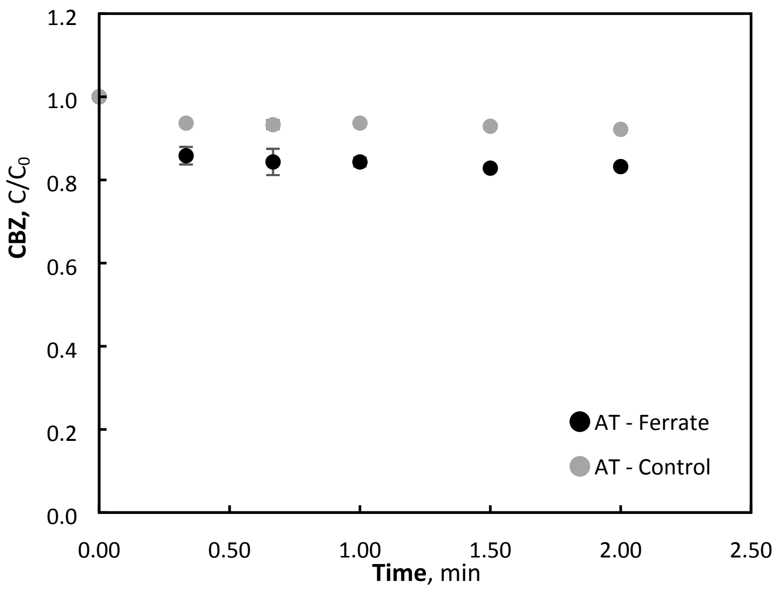

Figure S22. CBZ degradation using AT derived ferrate(IV/V) and NaCl control solutions (CBZ_0 = 10 μM).

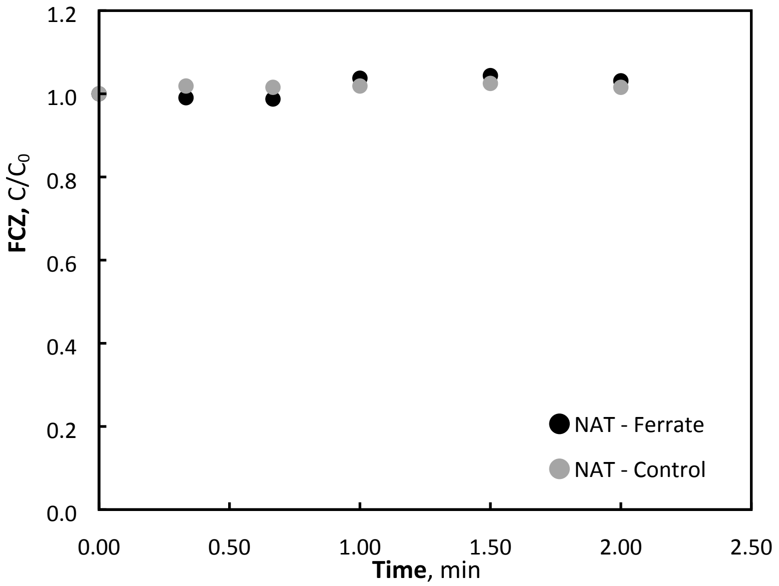

Figure S23. FCZ degradation using NAT derived ferrate(VI) and NaCl control solutions (FCZ_0 = 10 μM).

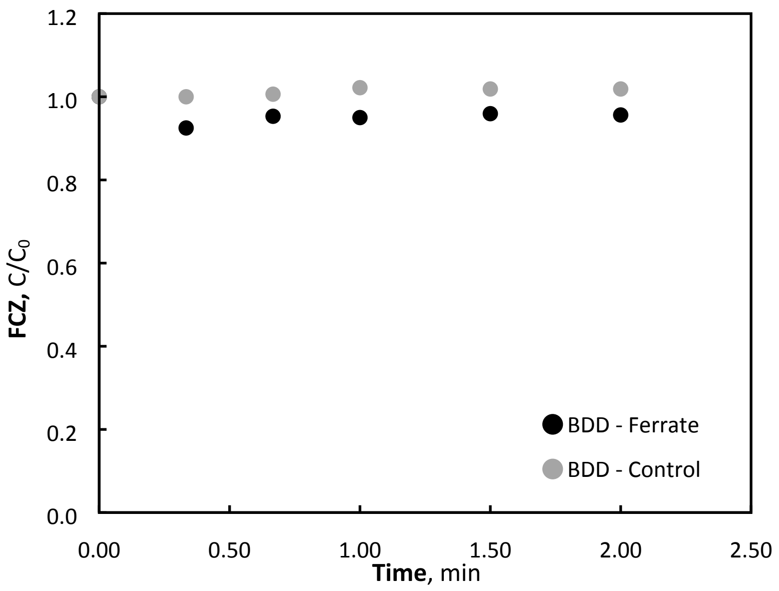

Figure S24. FCZ degradation using BDD derived ferrate(IV/V) and NaCl control solutions (FCZ_0 = 10 μM).

S.4. Ferrate Stability

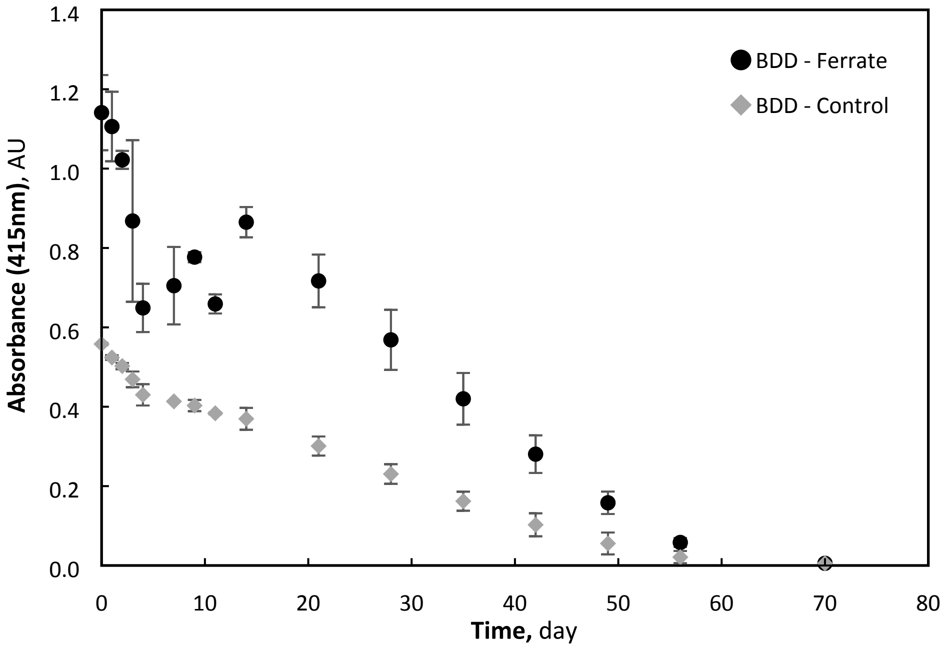

Figure S25. ABTS absorbance data for ferrate(IV/V) degradation long term study ($(Fe(VI))_{Eq}$ = 13.6 mM).

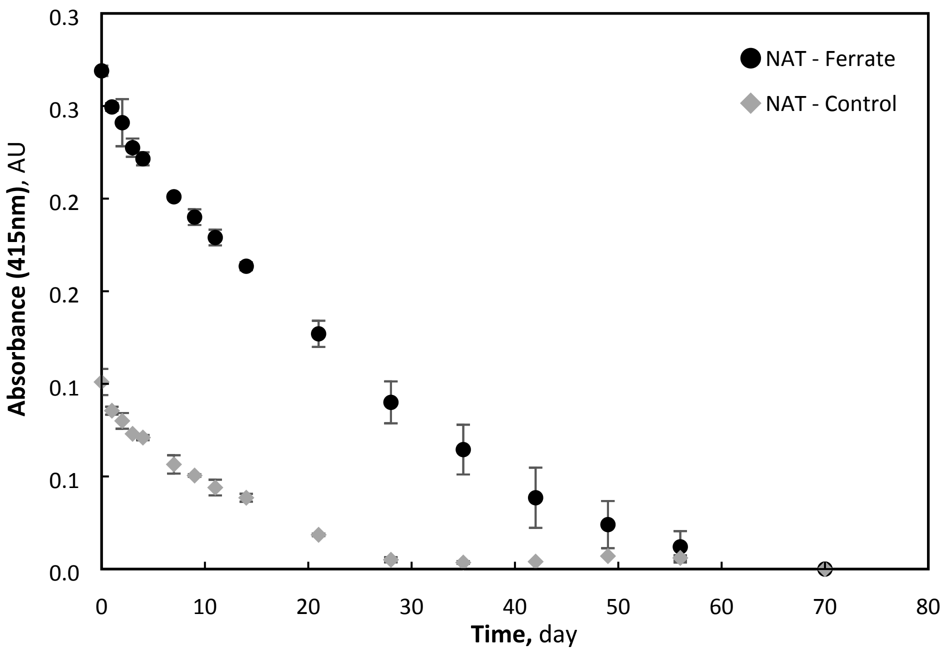

Figure S26. ABTS absorbance data for ferrate(VI) degradation long term study (Fe(VI) = 3.9 mM).

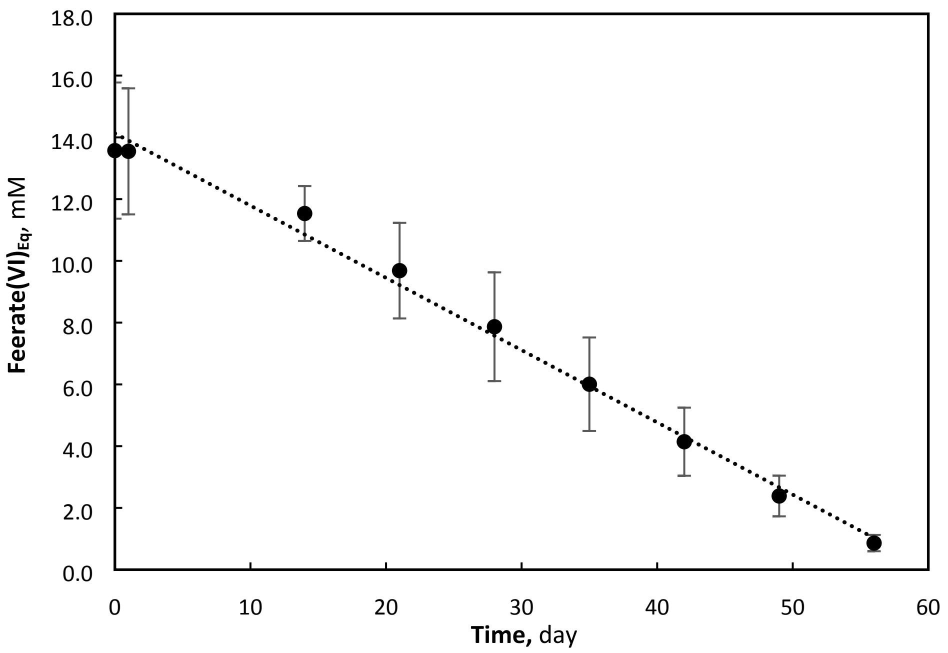

Figure S27. $Fe(VI)_{Eq}$ data for ferrate(IV/V) degradation long term study ($Fe(VI)_{Eq}$ = 13.6 mM). $Fe(VI)_{Eq}$ = Equivalent oxidative capacity of Fe(VI) with ABTS (1:1 molar ratio).

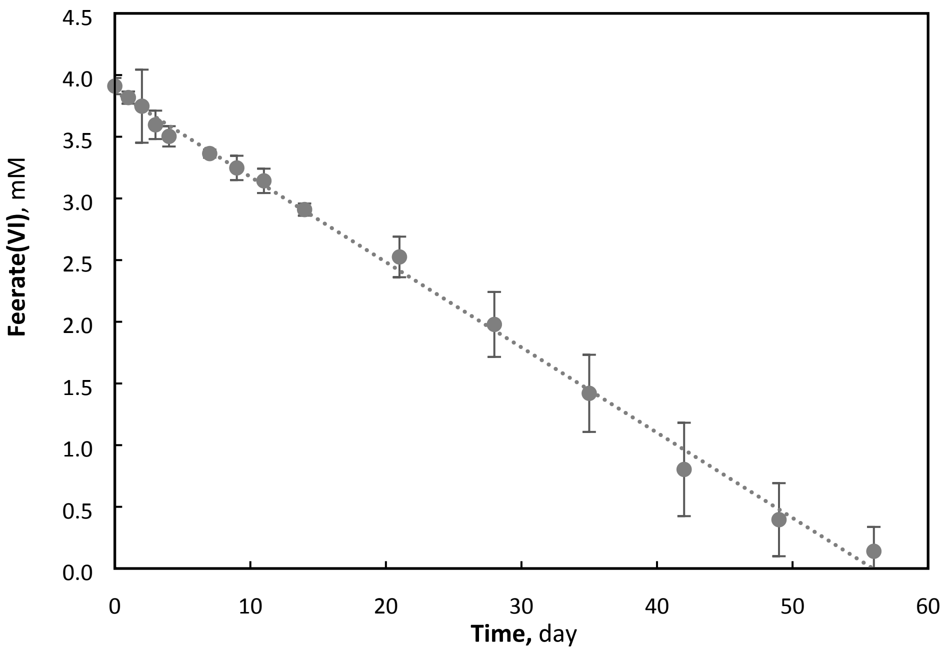

Figure S28. Fe(VI) data for ferrate(VI) degradation long term study (Fe(VI) = 3.9 mM).

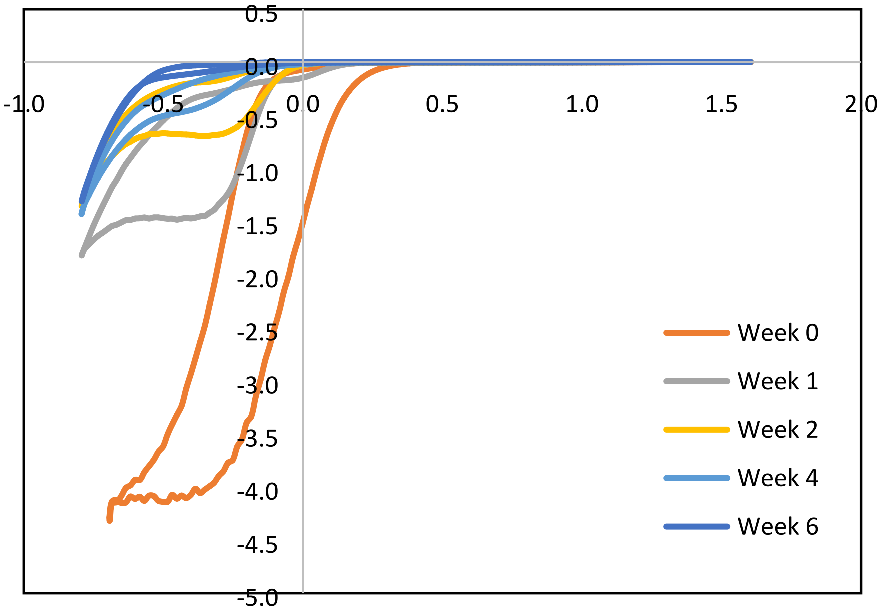

Figure S29. Cyclic voltammograms of BDD derived ferrate(IV/V) solution over 6 week stability test. CV condition: scan rate 50 mV s^{-1}, 0.0 V vs. E_{OC} to -1.3 V vs. ref.

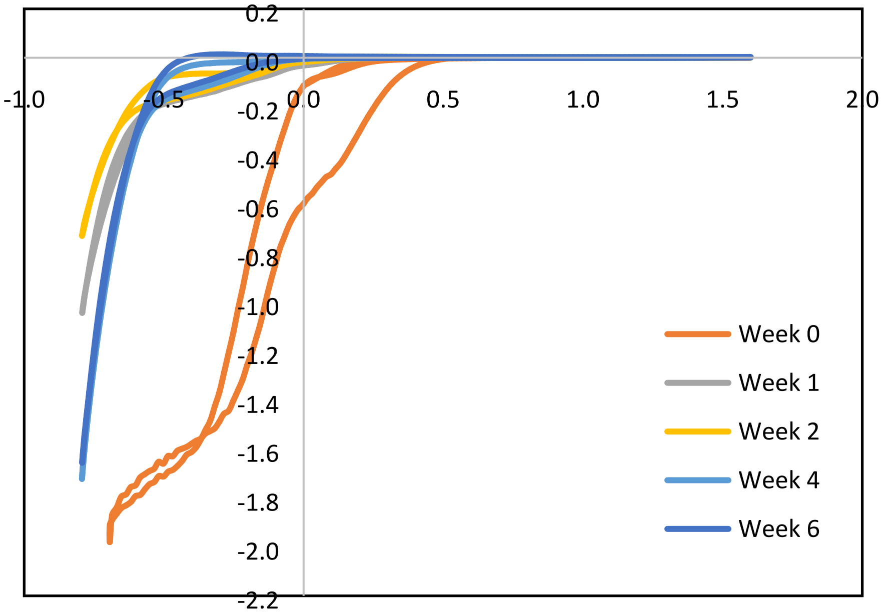

Figure S30. Cyclic voltammograms of NAT derived ferrate(VI) solution over 6 week stability test. CV condition: scan rate 50 mV s^{-1}, 0.0 V vs. E_{OC} to -1.3 V vs. ref.

S.5. Ferrate Generation Mechanism.

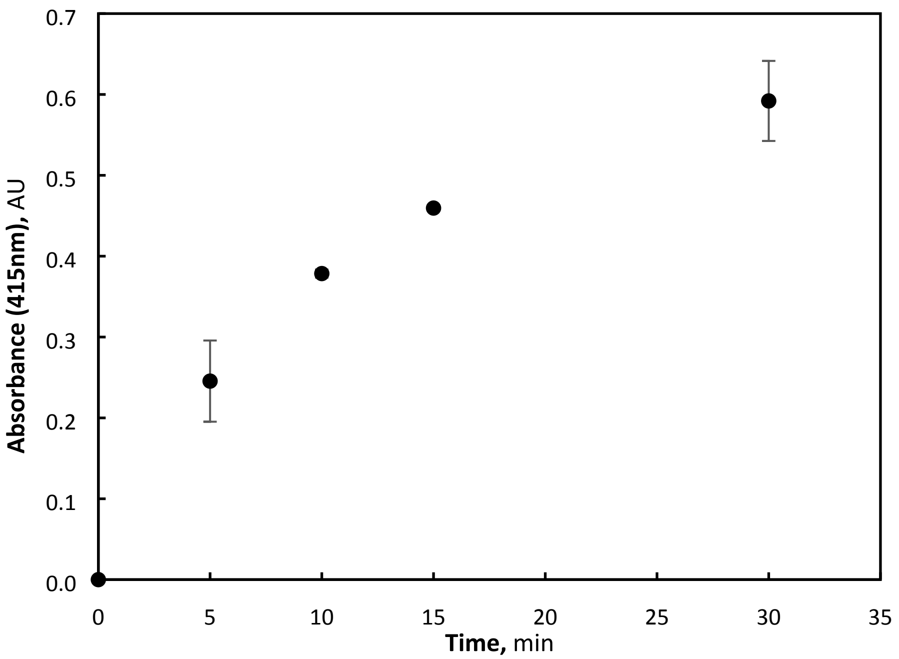

Figure S31. ABTS absorbance during BDD electrolysis and O_3 purging (10 mA cm^2, 15 mM $FeCl_3$).

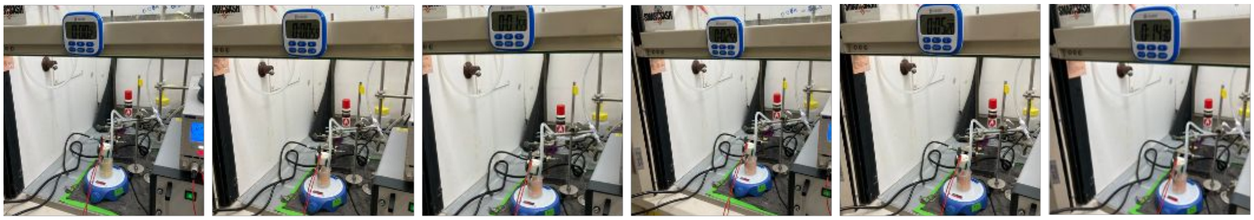

Figure S32. Ferrate(IV/V) oxidation experiments with O_3 during electrolysis to form ferrate(VI) (full set of photos).

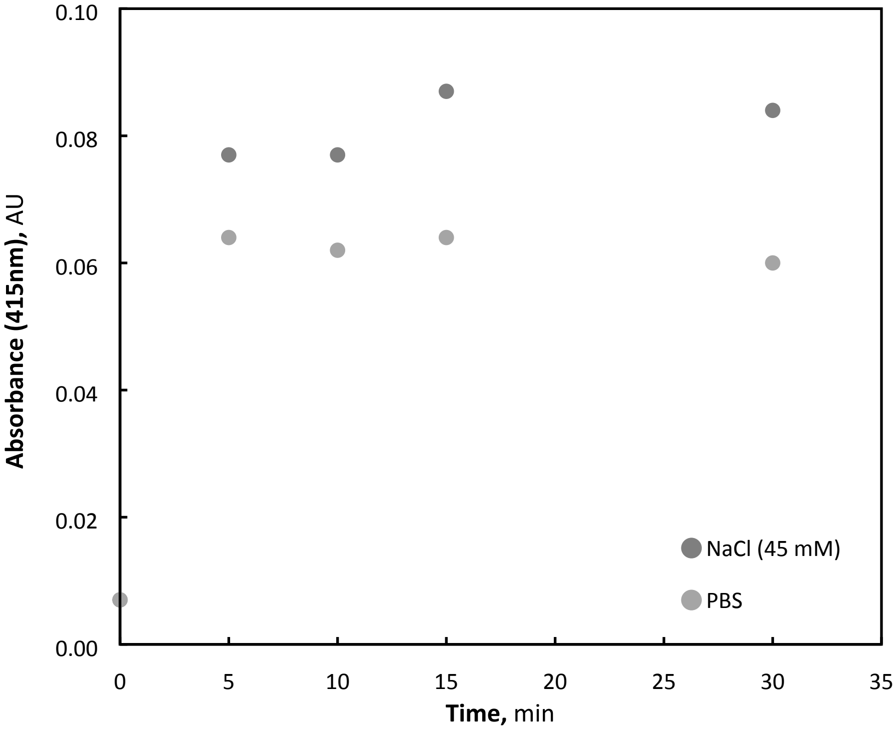

Figure S33. ABTS absorbance of control ozonation of NaCl and PBS solution.

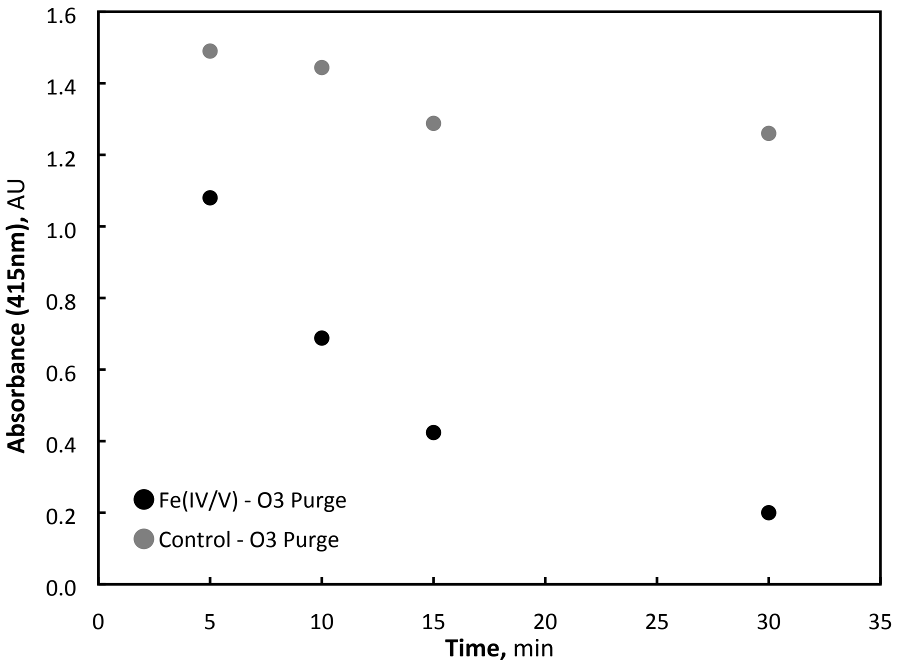

Figure S34. ABTS absorbance during O_3 purging on ferrate(IV/V) solution produced with BDD electrolysis (10 mA cm[2], 15 mM $FeCl_3$) and control (10 mA cm[2], 45 mM NaCl).

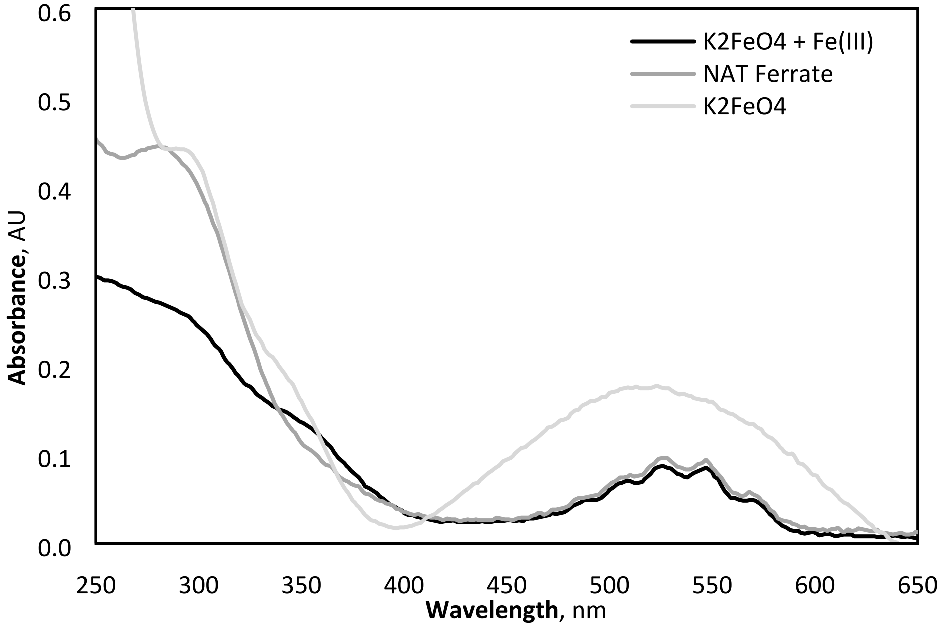

Figure S35. UV-absorption spectra of ferrate(VI) from K_2FeO_4, K_2FeO_4 and Fe(III) (dosed using $FeCl_3$) and NAT-generated ferrate(VI).
